# Chemoprevention of Colorectal Cancer by Dietary Compounds

**DOI:** 10.3390/ijms19123787

**Published:** 2018-11-28

**Authors:** Teodora Costea, Ariana Hudiță, Oana-Alina Ciolac, Bianca Gălățeanu, Octav Ginghină, Marieta Costache, Constanța Ganea, Maria-Magdalena Mocanu

**Affiliations:** 1 Department of Pharmacognosy, Phytochemistry and Phytotherapy, ”Carol Davila” University of Medicine and Pharmacy, 020956 Bucharest, Romania; teodora.costea@umfcd.ro; 2 Department of Biochemistry and Molecular Biology, University of Bucharest, 050095 Bucharest, Romania; arianahudita@yahoo.com (A.H.); bianca.galateanu@gmail.com (B.G.); marietacostache@yahoo.com (M.C.); 3 Department of Biophysics, ”Carol Davila” University of Medicine and Pharmacy, 050474 Bucharest, Romania; oana.ciolac@yahoo.com (O.-A.C.); constanta.ganea@gmail.com (C.G.); 4 Department of Surgery, ”Sf. Ioan” Emergency Clinical Hospital, 042122 Bucharest, Romania; octavginghina@yahoo.com; 5 Department II, Faculty of Dental Medicine, “Carol Davila” University of Medicine and Pharmacy, 030167 Bucharest, Romania

**Keywords:** colorectal cancer, chemoprevention, dietary compounds

## Abstract

Colorectal cancer is one of the leading causes of death, and the third most diagnosed type of cancer, worldwide. It is most common amongst men and women over 50 years old. Risk factors include smoking, alcohol, diet, physical inactivity, genetics, alterations in gut microbiota, and associated pathologies (diabetes, obesity, chronic inflammatory bowel diseases). This review will discuss, in detail, the chemopreventive properties of some dietary compounds (phenolic compounds, carotenoids, iridoids, nitrogen compounds, organosulfur compounds, phytosterols, essential oil compounds, polyunsaturated fatty acids and dietary fiber) against colorectal cancer. We present recent data, focusing on in vitro, laboratory animals and clinical trials with the previously mentioned compounds. The chemopreventive properties of the dietary compounds involve multiple molecular and biochemical mechanisms of action, such as inhibition of cell growth, inhibition of tumor initiation, inhibition of adhesion, migration and angiogenesis, apoptosis, interaction with gut microbiota, regulation of cellular signal transduction pathways and xenobiotic metabolizing enzymes, etc. Moreover, this review will also focus on the natural dietary compounds’ bioavailability, their synergistic protective effect, as well as the association with conventional therapy. Dietary natural compounds play a major role in colorectal chemoprevention and continuous research in this field is needed.

## 1. Introduction

The alarming incidence of colorectal cancer (CRC) has led to a pressing demand in developing novel therapeutic strategies that could overcome the limitations of conventional therapies. Depending on the cancer stage, its treatment usually involves surgery, radiation and chemotherapy. Because CRC is generally diagnosed in the late stages, when patients frequently present with distant metastases, chemotherapy represents the backbone of CRC treatment [1,2]. Despite the extensive use of chemotherapeutical agents, such as fluorouracil, oxaliplatin or irinotecan, chemotherapy presents several disadvantages like severe toxicity associated with serious adverse reactions, development of drug resistance, and a lack of specificity in targeting solely tumor cells [3,4].

Reports that sustain the beneficial effects of natural compounds administration for a broad spectrum of diseases associated with the numerous sources available for compound isolation have opened an emerging interest in using natural compounds as novel therapeutic candidates for CRC treatment. Natural compound based CRC therapy can be used through the entire process of CRC management. A favorable diet plan can prevent the disease based on a high consumption of vegetables, fruits and fibers. It had been shown that natural compounds can target tumor cells after disease occurrence and prevent tumor recurrence or metastasis [5]. Moreover, one of the most important features of natural compounds remains their capacity to induce tumor cells sensitivity for chemotherapeutic agents after the development of drug resistance [6,7]. It has been reported that the dietary compounds, such as quercetin and curcumin, succeeded to overcome the multidrug resistance in several malignant cell lines [8,9,10]. This chemosensitivity potential of natural compounds favor their use as adjuvant therapy in conventional treatment protocols, but exclusively natural compound treatment can be also based on a different mechanism of action against tumor cells [8,11]. Therefore, natural compounds can exert anti-tumor effects due to their antioxidant capacity, ability to inhibit cellular growth and trigger tumor cell apoptosis or by modulating the metastatic cascade.

## 2. General Aspects of CRC

CRC is one of the leading causes of death and the third most diagnosed cancer in the world along with breast, prostate, lung, ovary, pancreas and bladder cancers [12,13]. The premalignant lesions associated with CRC are multiple aberrant crypt foci (AFC) which precedes the evolution of the adenomatous polyps [14,15]. Regarding the genomic instability, there are known three main pathways involved in the initiation and progression of CRC: (i) The chromosomal instability (CIN) pathway, (ii) global genome hypermethylation correlated with the shutdown of tumor suppressor genes, known as CpG island methylator phenotype (CIMP), and (iii) DNA microsatellite instability (MSI) phenotype [16,17,18].

Chronic inflammation, which is usually present in inflammatory bowel diseases (IBD), such as Chron’s disease or ulcerative colitis, is also a trigger for CRC development, especially for patients under 30 years. The molecular and cellular changes in chronic inflammation of the bowel may include alteration in cellular immunity, over-expression of cyclooxygenase (COX-2), activation of toll-like receptor 4 (TLR4), nucleotide-binding oligomerization domain-like receptors (NLRs), overexpression of vascular endothelial growth factor (VEGF) and genetic factors [19,20,21]. Inflammation in patients with CRC is associated with increased cellularity of Peyer’s patches, a lymphoid tissue associated with the small intestine [22].

It is well known that genetics, race, gut microbiota and environmental factors play a key role in the tremendous increase of CRC worldwide. Among genetic factors, mutations in the several genes (the homolog of Kirsten rat sarcoma proto-oncogene, *KRAS*; the homolog of rapidly accelerated fibrosarcoma proto-oncogene, *BRAF*) and chromosomal instability (manifested by frequent mutations) are involved in CRC etiology. Moreover, the polymorphism of several proteins is responsible for CRC incidence. In this category might be included nucleic acid binding protein 1 (NABP1, a protein responsible for DNA repair), laminin-1, cyclin D2, and transcription factors which target wingless-related integration site (Wnt)/β-catenin pathway [12,23,24,25].

In addition, there are patients that have a truly inherited predisposition for CRC development, those with familial adenomatous polyposis, Peutz-Jeghers syndrome, serrated polyposis syndrome and Lynch syndrome [26].

Gut microbiota is involved in tumor pathogenesis through the production of detrimental metabolites (polyamines, hydrogen sulfide and secondary bile acids), bacterially derived genotoxins, host defense modulation, inflammation, and oxidative stress [27]. Detrimental metabolites can cause direct DNA damage through DNA alkylation or induce chronic inflammation via interleukin 6 (IL-6) and tumor necrosis factor alpha (TNF-α) production [28,29]. Intestinal dysbiosis is common for CRC and it involves high levels of gut bacteria, such as *Fusobacterium nucleatum*, enterotoxigenic *Bacteroides fragilis*, adherent-invasive *Escherichia coli*, *Peptostreptococcus anaerobius*, *Enterococcus faecalis*, *Bilophila wadsworthia*, *Solobacterium moorei* and low levels of *Bifidobacterium* sp., *Roseburia* sp., *Blautia* sp., *Lactobacillus* sp. [29,30,31,32].

Intestinal dysbiosis, it is also characterized by defective production of short chain fatty acids, such as butyrate and acetic/propionic acids. Butyrate, produced by *Eubacterium rectale* and *Roseburia* sp., represents the energy substrate for epithelial cells of the colon, increases mucus production, raise the activity of antioxidant enzymes, and has anti-inflammatory properties. Acetic and propionic acids have been shown to inhibit the activation of nuclear factor kappa-light-chain-enhancer of activated B cells (NF-κB) and to stimulate the apoptosis of tumor cells [19]. Pathogenic bacteria are responsible for a chronic intestinal inflammatory state via the recognition of microorganism-associated molecular pattern by Toll-like receptor 4 (TLR4), that induce activation of T helper 17 cells and production of pro-inflammatory interleukin 23 (IL-23) [28]. Moreover, these harmful bacteria are responsible for the increased activity of bacterial enzymes, such as β-glucuronidase, β-glucosidase, azoreductase, nitrate-reductase, alcohol dehydrogenase involved in converting heterocyclic aromatic amines, polycyclic aromatic hydrocarbons and primary bile acids into active carcinogens. In addition, harmful bacteria decrease the production of mucins by goblet cells and synthetize phenols, cresols and nitroso-compounds [19].

Environmental factors which favor the increased incidence of CRC include: Alcohol, smoking [33], consumption of red meat [34], associated pathologies, such as diabetes, overweight, and obesity [35], exposure to different chemicals (aromatic hydrocarbons), radiation [29,36], physical inactivity [37,38] and diet [38,39]. Red meat is a source of iron porphyrin pigment, which is responsible for the induction of carcinogenesis through the formation of nitroso-compounds [34]. Furthermore, the meat intake was related to CRC due to several factors: High fat-diet that might stimulate carcinogenesis through insulin resistance or fecal bile acids, heterocyclic amines produced by cooking the meat at high temperatures, N-nitroso compounds from processed meat, such as sausages, hot dogs, bacon [40,41].

Under these circumstances, it was reported that an increased intake of fruits and vegetables along with the limitation of a western diet (rich in fast food products and sweets), folic acid and vitamin D supplementation have a positive impact towards the reduction of CRC incidence [39,42].

Hormonal status also plays an important role in colon carcinogenesis mainly in women. Post-menopausal women have a high incidence of CRC, probably due to loss of cell cycle regulatory properties mediated by estrogen ERβ receptors or an increase in ERα receptors [43].

Screening remains the golden standard for preventing CRC and it is usually applied for people above 50 years. Screening methods include stool-based tests, visualization (colonoscopy) and serology/tissue markers. To reduce mortality and to improve the prognosis of CRC, earlier detection of serum markers is critical. The common biomarkers used in serological screening methods may include carcinoembryonic antigen (CEA), seprase, soluble cytokeratin-19 fragment (CYFRA 21-1), osteopontin (OPN), ferritin, anti-p53 [44,45].

## 3. General Aspects Regarding the Dietary Compounds

Healthy eating strategies, with increased consumption of fruits, vegetables, cereal grains, edible macrofungi and a balanced intake of meat and high fat products, have a beneficial role in the prevention of chronic diseases, such as heart disease, diabetes, cancer, neurodegenerative diseases or stroke [22,46]. Dietary phytochemicals can be divided into several categories, such as phenolic compounds, carotenoids, iridoids, nitrogen containing compounds, organosulfur compounds, phytosterols, dietary fiber, essential oils, polyunsaturated fatty acids [22,47].

### 3.1. Phenolic Compounds

Phenolic compounds are one of the widely distributed secondary metabolites in plants kingdom that share a common characteristic, the presence of at least one aromatic ring hydroxyl substituted. They usually bound to other molecules, such as glycosides and proteins and are well known for their powerful antioxidant properties [48]. Phenolic compounds can be divided according to their chemical structure into flavonoid and non-flavonoid constituents. Flavonoids are water-soluble pigments with a C6-C3-C6 skeleton, which have been subdivided in flavones, flavonols, flavanones, flavanols, isoflavones, and anthocyanidins [49]. Non-flavonoids compounds include tannins, phenolcarboxylic acids (derivatives of hydroxybenzoic and hydroxycinnamic acids), lignans, stilbens and other compounds. Phenolic compounds are widely distributed in fruits, vegetables, coffee, black tea, green tea and wine [50].

### 3.2. Carotenoids

Carotenoids are terpenic fat-soluble pigments containing 40 carbon atoms that share a polyisoprenoid structure, a long conjugated chain of double bonds and with a symmetry around the central bond [51]. According to their chemical structures, carotenoids are classified as carotens (α-carotene, β-carotene, lycopen—which are linear hydrocarbons that are cyclized at one end or both ends of the molecule) and oxygenated carotenoids (xantophylls), such as lutein, zeaxanthin [52]. Other important carotenoids are crocetin and its ester crocin. Dietary carotenoids are associated with low cardiovascular risk [53], eye health [54], prevention of infectious disease, anti-cancer and anti-inflammatory effects [51].

### 3.3. Iridoids

Iridoids are a type of monoterpenoids that have a cyclopentanopyran backbone, and are typically found in plants, such as glycosides. One of the most studied iridoids is oleuropein extracted from olives, which was known for its protective activity against cardiovascular and metabolic diseases [55].

### 3.4. Nitrogen Compounds

Nitrogen compounds are characterized by the presence of nitrogen atoms and are classified as alkaloids (a group of secondary plant metabolites, biosynthesized from amino acids) and non-alkaloid derivatives, such as protoalkaloids, pseudoalkaloids, alkamides, lectines, cyanogenic glycosides etc. [56]. Nitrogen compounds are characterized by great structural diversity and diverse pharmacological effects, such as analgesic, anti-inflammatory, antitussive, anticancer, antimalarial etc. [57].

### 3.5. Organosulfur Compounds

Organosulfur compounds contain at least one atom of sulfur in their molecule and are found mainly in garlic and cruciferous vegetables [58,59]. These compounds are well-known for their antifungal, antibacterian, anti-parasitic, antiviral, antithrombotic, immunomodulatory and anticancer effects [59].

### 3.6. Phytosterols

Phytosterols are plant derived lipid compounds, which resemble cholesterol, but vary in carbon side chains or presence/absence of a double bound. They are classified as sterols, (unsaturated compounds) and stanols (saturated molecules). Phytosterols are usually found in plants or macro fungi and share a wide range of therapeutic effects, such as hypolipidaemic, anti-inflammatory or anticancer effects [60].

### 3.7. Essential Oil Compounds

Essential oils are complex mixtures of chemical compounds with great structural diversity. Monoterpenes, sesquiterpenes and aromatic compounds are the main classes of bioactive substances found in essential oils. They have a wide spectrum of pharmacological effects, such as antibacterial, antifungal, antiviral, anti-mutagenic, cancer preventive and anti-inflammatory properties. Aromatic plants and spices (rosemary, fennel, coriander, thyme, cinnamon etc.) are excellent sources of essential oils [61,62].

### 3.8. Polyunsaturated Fatty Acids (PUFA)

Fatty acids are lipophilic compounds, for which the carbon chain can vary from two to 40 carbon atoms. They are essential components of membrane phospholipids. Fatty acids are usually classified as short chain (up to six carbon atoms), medium chain (eight-twelve carbons) and long chain (above 12 carbons). In addition, fatty acids are classified according to the presence or the absence of the double bonds in their molecules [63]. Long chain PUFA are categorized in omega-3 (n-3) and omega-6 (n-6) depending on the position of the first double bond from the methyl group of the fatty acid [64]. Humans are not able to synthesize the essential omega-3 fatty acids (α-linolenic acid) and omega-6 fatty acids (linoleic acid), so they depend exclusively on dietary sources [65]. Other omega-6, omega-3 fatty acids, such as γ-linolenic acid (GLA), di-homo-γ-linolenic acid (DHGLA), docosahexaenoic acid (DHA), eicosapentaenoic acid (EPA), arachidonic acid (AA) are generated during PUFA production, under the influence of different enzymes: ∆6 desaturase, elongase etc. [66]. The ratio between n-6/n-3 is very important for a balanced synthesis of eicosanoids [66,67] and the ideal ratio is 1/5–10 [66]. Recent findings support the role of PUFA in cardiovascular, neurodegenerative diseases or depression [64,68].

### 3.9. Dietary Fiber

Dietary fibers are found in fruits, vegetables, grains and macro fungi. They include non-starch polysaccharides, resistant starch fructo-oligosaccharides, galacto-oligosaccharides, glucomannans, lignin, pectin and β-glucans [63,69,70]. This large category of natural compounds has been classified according to their solubility, their quality of being fermentable and their physiological effects. Soluble fiber dissolves in water and form gels. They consist of inulin, pectin, gums and β-glucans. Insoluble fibers are represented by lignin, cellulose and some hemicelluloses [63]. Dietary fiber has various health benefits, such as lowering cardiovascular risk, glycemic and body weight control, laxation, prebiotic effect [63,69,71].

Some of the most well-known dietary compounds, belonging to different categories are presented in Table 1 (phenolic compounds) and Table 2 (non-phenolic compounds), including members, chemical structures and dietary sources.

## 4. In Vitro Studies

### 4.1. Polyphenols

**Figure 1:**
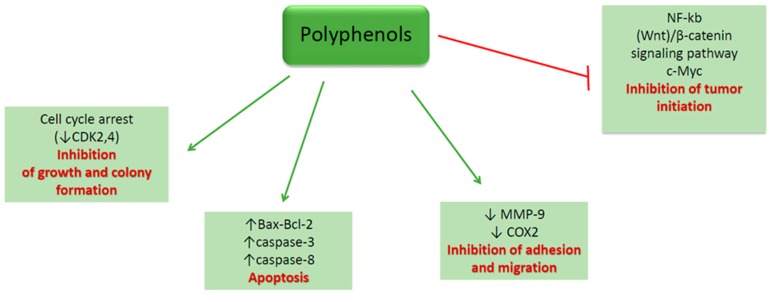
Schematic representation of the polyphenols’ major mechanisms of action on colorectal cancer (CRC) cells. Polyphenols: (i) Determine cell cycle arrest by downregulating cyclin-dependent kinase 2, 4 (CDK 2, 4), (ii) induce apoptosis by upregulating B cell lymphoma 2 associated protein X and B cell lymphoma 2 (Bax-Bcl-2), caspase-3 and caspase-8, (iii) inhibit cell adhesion and migration through the downregulation of matrix metalloproteinase 9 (MMP-9) and cyclooxygenase 2 (COX2) and (iv) stop tumor initiation by altering nuclear factor kappa-light-chain-enhancer of B cells (NF-kb)/β-catenin signaling pathway.

The potential use of polyphenols for CRC prevention and treatment had been widely investigated (Figure 1). Research results highlight the potential of the dietary bioactive compounds to interfere with tumorigenesis at all steps tumorigenesis, including initiation, promotion and progression. In vitro mechanisms of dietary polyphenols demonstrated their ability to modulate cellular processes, such as, gene expression, apoptosis or differentiation [80]. Several recent studies have demonstrated that cancer treatment through a combinatorial approach might show increased efficiency than the use of drugs only [81].

#### 4.1.1. Flavones

*Quercetin* is a flavonol present in vegetables and fruits like as onion or apples. Quercetin reduces proliferation in CRC cell lines by downregulating RAS p21 protein activator 1 gene [82]. The combination of quercetin with tumor necrosis factor-related apoptosis-inducing ligand (TRAIL) proved a TRAIL-induced apoptosis in a synergistic manner in colorectal adenocarcinoma cells (SW-620 and HT-29 cell lines), while this combination resulted in an additive effect in the case of Caco-2 human epithelial colorectal adenocarcinoma cells [83]. Quercetin has been also tested in vitro in combination with fluorouracil (5-FU) in CO115 human colon carcinoma cells (p53 positive) and HCT15 colorectal adenocarcinoma cells (p53 negative). This combination of drugs showed higher apoptosis levels in CO115 cell line, in a synergistic manner, but as an additive effect in HCT15 cells [84].

*Kaempferol* can be found in black tea, broccoli, propolis or grapefruit acts on different types of cancer cells [85] inducing apoptosis through cytochrome c mitochondrial release, caspase-3 cleavage activation and p53-dependent growth inhibition [86,87]. Cho et al. showed in 2012 that kaempferol may induce G1 and G2/M cell cycle arrest by inhibiting the activity of cyclin-dependent kinase 2, 4 (CDK 2, 4) and cell division cycle protein 2 [88]. In HT-29 cells, kaempferol induces apoptosis and inhibits insulin-like growth factor 1 receptor (IGF-1R) and receptor tyrosine kinase ErbB3 signaling pathways [89]. Kaempherol was tested in combination with TRAIL on SW480 and DLD-1 CRC cell lines and the results showed an increase in apoptotic induction in a kaempferol-dose dependent manner, probably by up-regulating death receptor-5 [90].

*Apigenin* is one of the well-known flavones which is present in several fruits and vegetables, such as parsley, garlic, Chinese cabbage, celery, bell pepper, and guava [72]. It is a chemopreventive agent with strong cytostatic and anti-angiogenic effects in vitro [72]. In CRC cells, the activity of apigenin was correlated with a blockage in cell cycle progression, induction of apoptosis and inhibition of the cell growth [91,92,93]. However, to increase the anti-neoplastic activity of apigenin additional combination therapy with drugs, such as 5-FU, oxaliplatin or irinotecan, might be required.

#### 4.1.2. Isoflavones

*Genistein* is an isoflavone, which can be found in high concentrations in soybeans, lentils, beans, and chickpeas. A negative correlation was reported between the soybean diet and the occurrence of the CRC [94,95]. This isoflavone aroused a growing interest as a pro-apoptotic agent because of succeeded to be more effective in CRC cells compared to their normal counterparts [96]. Genistein acts by increasing the expression of B cell lymphoma 2-associated X protein (Bax) or cyclin-dependent kinase inhibitor, p21 [97], by inhibiting Nuclear factor kappa-light-chain-enhancer of activated B cells/NF-κB [98] and topoisomerase II [99]. In addition, genistein displayed anti-cancer activities by regulating ErbB proteins expression [100] and by suppressing the carcinogen induction of Wnt/β-catenin signaling pathway [101]. In addition, genistein was tested together with 5-FU on HT-29 cells and the results showed a synergistic effect on cell growth blocking, probably due to the over-expression of pro-apoptotic p53 and p21 and down-regulation of COX-2 [102].

#### 4.1.3. Phenocarboxilic Acids

*Caffeic acid* showed a pro-apoptotic effect on HT-29 cells [103,104] starting from 80 μM concentration, while *chlorogenic acid* [105] did not show any significant activity against different human colorectal carcinomas [106]. *Ferulic acid* displayed inhibition of CRC progression acting on cells adhesion and migration mechanisms [103]. *Gallic acid* is the only one among benzoic acid derivatives that acts on CRC cells by upregulating Bax and downregulating Bcl-2 [107,108].

#### 4.1.4. Stilbens

*Resveratrol* is a phytoalexin found in many plant species, including edible plants like grapes or berries. Resveratrol is synthesized by plants in response to environmental stress and pathogenic invasion as a natural inhibitor of cell proliferation [81]. The first study revealing the resveratrol’s chemopreventive activity was published by Jang et al. in 1977, who described the effect of the topical administration of resveratrol [109]. Since then, resveratrol was used in many studies for various malignancies, including CRC. In vitro studies demonstrated that resveratrol might modulate and inhibit tumorigenesis by reduction of the COX-2 and cytochrome P450 activity. Moreover, resveratrol can exert synergistic activities in combination with other active compounds, such as quercetin or chemotherapeutic agents, such as 5-FU, oxaliplatin or mitomycin C [110].

Consequently, the combination of resveratrol and quercetin stimulated apoptosis in HT-29 cell line, by reducing the RNA and proteins levels for several transcription factors [111]. This combination of the natural compounds suppressed human colon cancer stem cells proliferation and down-regulated c-Myc [112]. The toxicity of 5-FU was increased in CRC cell lines by synergistic activity of resveratrol. The mechanism of action of the combined treatment included an increase in the oxidative stress associated with inhibition of Akt and STAT3 pathways [113]. In addition, resveratrol together with 5-FU significantly induced apoptosis and reduced migration in CRC cells [114].

Other recent studies show that resveratrol chemosensitizes HT-29 and HCT-116 cells to oxaliplatin by upregulating miR-34c [115]. In addition, resveratrol and oxaliplatin synergistically inhibited cell growth of Caco-2 cells via apoptosis and necrosis induction [116]. At the same time, resveratrol can induce p21WAF1/CIP1 overexpression regardless of p53 status, and a combined treatment of resveratrol and mitomycin C repressed the proliferation of mitomycin C-resistant CRC cells [117].

#### 4.1.5. Other Compounds

*Curcumin*, a diarylheptanoid found in turmeric, was identified as a chemopreventive dietary compound in CRC [118,119,120]. Curcumin showed a synergistic effect with dasatanib, a potent inhibitor of Src and Abl kinases, in CRC cell lines with resistant phenotype to FOLFOX (folinic acid, fluorouracil, oxaliplatin) chemotherapy [120]. The anti-neoplastic effect of 5-FU was reported to be increased by curcumin [121,122]. Curcumin can potentiate, as well the pro-apoptotic and anti-metastatic effects of capecitabine [123].

### 4.2. Non-polyphenolic Compounds—In Vitro Mechanism of Action

**Figure 2:**
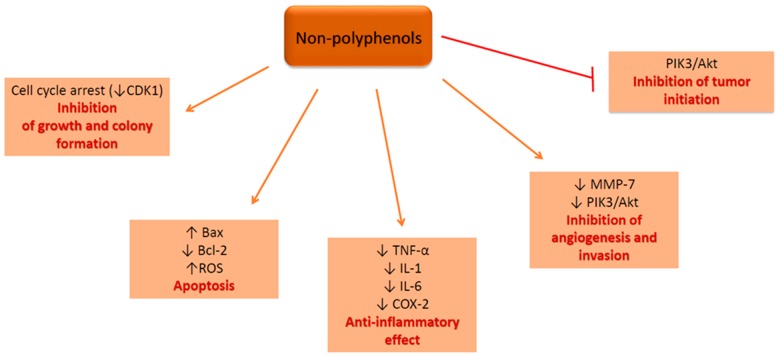
Schematic representation of the non-polyphenolic compounds’ major mechanisms of action on CRC cells. Non-polyphenolic compounds: (i) Determine cell cycle arrest by downregulating cyclin-dependent kinase 1 (CDK 1), (ii) induce apoptosis by upregulating B cell lymphoma 2 associated protein X (Bax), B cell lymphoma 2 (Blc-2) and by increasing the production of reactive oxygen species (ROS), (iii) exert anti-inflammatory effects by downregulating interleukin 1 (IL-1), interleukin 2 (IL-2), interleukin 6 (IL-6) and cyclooxygenase 2 (COX2), (iv) inhibit angiogenesis and invasion by downregulating matrix metalloproteinase 7 (MMP-7) and phosphoinositide 3-kinase (PKI3)/Protein kinase B (Akt) and (v) stop tumor initiation by altering the PKI3/Akt pathway.

Non-polyphenolic compounds display chemopreventive effects on CRC cells in vitro modulating signaling pathways with impact on cell cycle arrest, apoptosis, invasion, inflammation and much more (Figure 2).

#### 4.2.1. Carotenoids

Even if the benefits of a *lycopene* diet in CRC prevention have not been clearly demonstrated [124], lycopene remains a potential chemopreventive agent for CRC management due to its excellent antioxidant capacity [125]. Lycopene inhibits the cellular growth of HT-29 cells in a dose-dependent manner by effectively inhibiting the phosphorylation of Protein kinase B (Akt) and therefore silencing Phosphatidylinositol-4,5-bisphosphate 3-kinase (PIK3)/Akt, a signaling pathway that is associated with colorectal tumor development [126]. Moreover, lycopene suppresses tumor cell invasion mediated by leptin as proteomic assays revealed that lycopene exposure significantly decreased expression levels of MMP7 and thereby reduce the tumor cells invasion capacity [127]. Tanga et al. highlighted a synergistic mechanism between lycopene and eicosapentaenoic acid that significantly increased expression of proapoptotic molecules Bax and Fas. Eicosapentaenoid acid and lycopene reduce tumor cell growth also by suppression of PIK3/Akt pathway and by further blocking the activation of downstream molecule mTOR [128]. Another promising effect of lycopene use as a chemopreventive agent was based on its anti-inflammatory potential against different inflammation-related proteins associated with CRC development [129]. In SW480 CRC cell culture, lycopene modulates the inflammatory cascade by inhibiting the protein expression of NF-κB and JNK. In human CRC cells SW480, after the stimulation with lipopolysaccharides, the level of inflammatory cytokines (TNF-α, IL-1 and IL-6) as well the inflammation-inducing enzymes (COX-2 and iNOS) was reduced when treated with lycopene. Consequently, this compound shows promising effects for modulating the NF-κB signaling pathway in CRC associated inflammation [130].

*β-carotene* significantly reduce in vitro cell proliferation of CRC lines LS180, SW620 and HCT-15 and its cytotoxic potential can be easily tailored by adjusting different experimental parameters, such as dose, exposure time and cell seeding density [131]. However, the cytotoxic potential of β-carotene was strongly affected by the cell capacity to incorporate the carotenoid as different cell lines present various patterns of growth inhibition in presence of the same concentration of compound [132]. Briviba et al. associated β-carotene exposure of HT-29 cells with induction of apoptosis through a mechanism independent expression of the MEK/ERK signaling pathway as the expression of extracellular signal regulated kinases ERK1 and ERK2 was not affected [133]. The anti-cancer potential of β-carotene was attributed to cell cycle arrest and apoptosis induction by modulating the expression of different key regulator proteins. In the presence of β-carotene, COLO320HSR cells exhibit cell cycle arrest in G2/M phase mediated by the down-regulation of cyclin A expression and apoptotic death correlated with decreased levels of apoptotic inhibitors Bcl-2 and Bcl-xL [134]. β-carotene modulates also the apoptotic pathway mediated by Bcl-2 through its pro-oxidant activity, as it is capable to stimulate ROS production in CRC lines [135].

*Crocetin* is a promising chemopreventive agent for CRC mainly due to its antioxidant and anti-inflammatory potential [136]. Crocetin extracts have proven to be effective in inhibiting the cellular growth of HT-29, HCT-116 and SW480 CRC cell lines in a dose-dependent manner [137]. Even if several mechanisms of action have been described for crocetin use in different types of cancer, the role of crocetin in CRC development inhibition has not been fully elucidated [138,139]. Crocetin promotes apoptosis via p53 dependent or independent mechanisms, increasing the attraction of crocetin use in defective p53 tumors [140,141]. Moreover, HTC-116 colon cancer cells treated with crocetin presented a significant reduction in the expression of inflammation related genes like IL-6, IL-8 and High mobility group box 1 protein (HMGB1), associated with an increased expression of NAG-1 gene that encodes a protein with high anti-tumorigenic activity [142].

#### 4.2.2. Nitrogen Compounds

*Piperine*, the most common dietary alkaloid was intensively used in clinics based on its strong antioxidant and anti-inflammatory capacity, but various studies revealed the great potential of using this natural compound as an active anti-neoplastic agent. Piperine specifically inhibits tumor cells proliferation and arrest cell cycle in G1 phase as revealed after HT-29 cells exposure to piperine. The cell cycle lock is commonly associated with a decreased expression of cyclins D1 and D3, up-regulation of p21WAF1 and p27KIP1 expression and a diminished phosphorylation of Rb protein [143]. Piperine mediated apoptosis can be activated as a result of ROS production increase [144] or by modulating mTORC1 signaling cascade as Caco-2 and HT-29 cells exposure to piperine is associated with inhibition of mTORC1, a key regulator of cellular autophagy [145].

*Capsaicin* is a promising natural agent in CRC therapy as various favorable effects on different CRC cell lines have been reported regarding the capacity of capsaicin to suppress tumor cell expansion [146,147,148,149]. However, in vitro capsaicin administrated doses need to be tightly adjusted since low concentrations of capsaicin promote metastasis by interfering with key molecules involved in the metastatic cascade. At low concentrations, capsaicin favors EMT transition by inhibiting E-cadherin expression, sustains tumor cell migration by inducing over-expression of MMP2 and MMP9 and activates Akt/mTOR signaling pathway, molecular events that together enhance the migration and invasive potential of SW480 cells. In contrast, at proper concentrations capsaicin is effective in reducing the metastatic burden by inhibiting overproduction of MMPs and the epithelial mesenchymal transition [150]. One of the main molecular mechanisms of action that underlies the role of capsaicin in cell proliferation inhibition is cell cycle arrest and subsequent induction of apoptosis mainly by modulation of p53 activity and other apoptosis linked molecules [151]. Colorectal tumor apoptotic cells are characterized by AMP-activated protein kinase (AMPK) signaling cascade activation [152], activation of the pro-apoptotic caspase-3 [149] and an altered expression pattern of β-catenin and transcription factor 4 (TCF-4) that blocks their interaction [153]. Apoptosis can be orchestrated by NO levels that trigger apoptotic pathways dependent or independent of p53 activity or by ROS levels that generate an impairment of the mitochondrial membrane potential [149,154]. Additionally, capsaicin also exhibits an efficient immunomodulatory effect as reveled by a dose-dependent decrease of numerous inflammatory cytokines after capsaicin treatment of HT-29 and RKO tumor cells [155]. Capsaicin presents a superior anti-tumor effect in combination with 3,3′-diindolylmethane by modulating the transcriptional activity of p53 and NF-κB together with other apoptosis related genes [156].

In conclusion, recent in vitro studies reveal that polyphenols act on colon cancer cells by inducing apoptosis and by inhibiting cells growth, migration, adhesion and tumor initiation through Wnt/β-catenin signaling pathway. Non-polyphenolic compounds induce apoptosis and inhibit cell growth, inflammation, angiogenesis and tumor initiation through PIK3/Akt signaling pathway. Considering the evidence provided by the in vitro studies regarding the mechanisms of action of the dietary polyphenols and non-polyphenolic compounds in CRC cells, further investigations should focus on underlying their efficacy and safety use in combination with chemotherapy and/or radiotherapy.

## 5. *In Vivo* Studies in CRC Animal Models

### 5.1. The Effect of Phenolic Compounds

The summary of the effects of phenolic compounds is presented in Table 3.

#### 5.1.1. Isoflavones

In vivo studies revealed that *genistein* treated high-fat mice with CRC induced by administration of azoxymethane/dextran sulfat sodium decreased the expression of inflammatory factors [157]. *Genistein* has also shown tumor suppressive activity in mice colon cancer through apoptotic effects, reduction of tumor weight and reduction of wingless-related integration site (Wnt) signaling [101,158]. Genistein was tested on BALB/c mice bearing and CT26 xenografts under radiotherapy and the results showed less non-tumorigenic apoptotic cells and improved morphological changes in healthy intestinal tissue [158]. More than 70% of patients with CRC undergoing radiotherapy displayed side effects at the gastrointestinal level. Normal colonocytes might be frequently damaged mainly as a result of an increased oxidative stress induced by radiation therapy, resulting in inflammatory or ulcerative lesions [110]. Administration of natural compounds as radioprotective mediators might represent a possible solution to avoid the occurrence of the mucosities, as side effects of the ionizing radiation therapy [172]. Xenografts’ mice with colon cancer tumors are treated with 5–10 Gy, and genistein was administrated one day prior to irradiation. The authors reported that genistein protected against intestinal injury induced by radiation therapy in mice [158].

#### 5.1.2. Anthocyanidins

Regarding administration of anthocyanidins, *cyanidin* from tart cherries significantly reduced adenomas of the cecum [159]. Shi N. and coworkers (2015) investigated the effect of dietary lyophilized strawberries, which are known to be rich in cyanidin and *pelargonidin glycosides*, on colon carcinogenesis induced by administration of azoxymethane. The results are promising with inhibition of tumor development associated with reduced phosphorylation of PI3K/Akt, declining levels of NF-κB and decreased expression of pro-inflammatory markers [160]. Recent researches have shown that administration of grape juice concentrate rich in phenolic compounds (*peonidin glucoside*, *malvidin-glucoside*) reduced inflammation, through decreased expression of COX-2, if taken before or after induction of colon cancer [161]. Other authors reported that administration of anthocyanin-rich diet in an animal model of CRC showed significant reduction of colon tumors and a positive effect on gut microbiota [162].

#### 5.1.3. Phenocarboxilic Acids

Phenolcarboxylic acids, *chlorogenic acid* showed chemopreventive effects in a mouse model of CRC [163]. Banerjee N. and coworkers demonstrated that administration of a plum beverage rich in phenolic compounds (chlorogenic and neochlorogenic acid) decreased the number of aberrant crypt foci, modulated the activity of several intracellular payhways and reduced inflammation [164]. *Cinnamic acid* upregulated the expression of protein Bax, a pro-apoptotic protein, in colorectal xenografts grown in athymic mice, probably by inhibition of histone deacetylases [165]. *Gallic acid*, *rosmarinic acid* and *p-coumaric acid* have a chemopreventive effect colon carcinogenesis induced by 1,2-dimethyl hydrazine induced. The phenolcarboxylic acids decreased tumor incidence, induced the inhibition of precancerous lesions, reduced gut bacterial enzymes (mucinase, nitroreductase, sulphatase, β- glucosidase) and displayed antioxidant properties [166,167,168]. It is well known that phase I metabolizing enzymes are required for activation of different carcinogens into forms capable of binding to proteins or DNA and leading to mutations. An increase in phase II metabolizing activity has been related to the elimination of carcinogens, through the formation of hydrophilic compounds, such as glucoronides, glutathione conjugates or glutathione sulphates [167]. Moreover, the phenolcarboxylic acids significant increased phase II xenobiotic metabolizing enzymes (glutathione S-transferase, gamma-glutamyl-transpeptidase) and decreased phase I xenobiotic metabolizing enzymes, such as cytochrome P450 and cytochrome b5 [166,167,168].

#### 5.1.4. Lignans

Lignans have strong anticancer effects against CRC; administration of a rich *secoisolariciresinol* extract from flaxseeds, reduced cancer biomarker levels and decreased the number of the proliferating cells [169]. Moreover, deflated flaxseeds reduced the incidence of precancerous lesions in the proximal, distal and middle colon. The exact mechanism of action is not known, since no changes are observed in tumor protein p53 (p53), cyclin-dependent kinase inhibitor 1 (p21^CIP1/WAF1^) and multiple tumor suppressor 1 (p16) expression and the authors did not notice an increase in fecal short chain fatty acids [170]. *Sesamol*, a lignin from sesame seeds, significantly reduced the number of intestinal polyps in mice with colorectal cancer, through suppression of COX-2 and prostaglandin E2 receptor expression levels [171].

### 5.2. The Effect of Non-Phenolic Compounds

The summary of *in vivo* experiments regarding CRC and non-phenolic compounds is presented in Table 4.

#### 5.2.1. Carotenoids

The effect of carotenoids (*lycopene*, *crocin*) on CRC was observed using animal models. Results are encouraging, since both dietary products showed chemopreventive effects and reduced the incidence of pre-neoplasic polyps, through various mechanisms, such as up-regulation of p21^CIP1/WAF1^ proteins, suppression of proliferating cell nuclear antigen (PCNA) expression and nuclear levels of β-catenin in tumor tissues. Moreover, an anti-inflammatory effect has been seen through suppression of COX-2 and prostaglandin E2 (PGE2) gene expression. Lycopene also inhibits matrix metallopeptidase protein (MMP-9) in correlation with reduction of angiogenesis, tumor invasion and metastasis. Crocin reduced the chronic inflammation by inhibition of the activated nuclear factor NF-κB and displayed a protective role regarding the toxicity of xenobiotics by increasing the level of Nrf2, nuclear factor (erythroid-derived 2)-like 2 [173,174,175].

#### 5.2.2. Iridoids

*Oleuropein*, an iridoid found mainly in olive leaves and fruits, can prevent CRC by regulation of Wnt/β-catenin, NF-κB, PI3K/Akt pathways, anti-inflammatory activity associated with significant decreased the intestinal concentrations of several interleukins (IL-6, IL-17), TNF-α and inhibited COX-2 activity. Besides oleuropein up-regulated Bax protein expression and induced apoptosis in intestinal tumor cells [176].

#### 5.2.3. Nitrogen Compounds

Pre-clinical studies have demonstrated that *capsaicin* may be a chemopreventive agent in CRC through anti-proliferative, anti-genotoxic, induced expression of apoptotic genes, and up-regulation of genes involved in cell differentiation [177]. Since previous reports indicated that capsaicin might promote metastasis, acting as co-carcinogen in cancer skin [196] and promoting breast cells metastasis [197]. Yang et al., investigated the correlation between capsaicin and metastasis in a tumor xenografts mouse model for CRC [150]. To investigate the ability of capsaicin to induce metastasis CT-26 murine CRC cell previous treated or not with 100 μM are intravenously injected in mice. The investigation of the pulmonary metastatic nodules in 15 days after injection validated the previous reports. Namely, the capsacin-treated cells increased the number of pulmonary metastatic nodules in mouse models [150]. Under these circumstances, caution should be taken when various doses of capsaicin are used for designing *in vivo* experiments or clinical trials, if recommended.

#### 5.2.4. Phytosterols

Among phytosterols, *β-sitosterol* has proved antioxidant activity in 1,2 dimethylhydrazine (DMH) colon carcinogenesis and restored endogenous antioxidant enzyme levels, such as superoxide dismutase, catalase, glutathione reductase, glutathione peroxidase and vitamins, such as vitamin E and C [179].

#### 5.2.5. Organosulfur Compounds

Down-regulation of histone deacetylase (HDAC) enzymes play a key role in cell cycle arrest and apoptosis, whilst up-regulation of Nrf2 expression is involved in the increased activity of phase II metabolizing enzymes [198,199]. *Sulforaphane*, from broccoli, inhibits CRC carcinogenesis by modulating Nrf2 activity and inhibition of HDAC enzymes [178].

#### 5.2.6. Essential Oils

Former data about the administration of essential oil compounds, mainly *thymoquinone* to male albino WISTAR rats with chemical induction of CRC reported a reduction in tumor incidence, volume and multiplicity and decrease in vascular endothelial growth factor (VEFG) concentration [180]. In addition, the association between thymoquinone and mesalazine, an anti-inflammatory drug, significantly reduced tumor incidence and multiplicity in transgenic mice [181]. Recent data reported that *cinnamaldehyde*, the main compound of cinnamon essential oil, induces Nrf2 activation in CRC tissues, without any toxicity [182]. *Carvacrol* is a monoterpenic phenol that occurs in essential oils of the *Labiatae* family (*Thymus sp.*, *Origanum sp.*, *Satureja sp.*). Administration of carvacrol to adult male albino rats with chemical induced CRC, revealed that carvacrol reduced the number of colon tumors, dysplastic polyps, aberrant crypts foci, increased glutathione peroxidase and glutathione reductase activities and restored levels of liver peroxidation products [183]. Moreover, combined treatment between carvacrol and X-radiation significantly decreased tumor incidence [200].

#### 5.2.7. Polyunsaturated Fatty Acids

Dietary feeding of polyunsaturated *omega-3 fatty acids* in C57 BL6 mice with CRC induced by inoculation of MC38 murine colon adenocarcinoma cells significantly inhibited the tumor growth. The possible molecular mechanisms included the augmentation in the levels of omega-3 metabolites (epoxydocosapentaenoic acids), suppression of inflammation through the reduction of the level of the pro-inflammatory agents (arachidonic acid eicosanoids), and decrement in the expression of human retroviral correspondent identified in myelocytomatosis, *MYC* protooncogene [184]. Nevertheless, more recent data reported that a ketogenic diet (low carbohydrates and high fat diet) with or without omega-3 fatty acids supplementation delayed tumor growth and suppressed tumor neovascularization [185]. Supplementation of diet with *eicosapentaenoic acid* in C57BL/6J mice with CRC induced by administration of azoxymethane suppressed tumor growth, increased apoptosis, and decreased systemic inflammation. In addition, supplementation with *eicosapentaenoic acid* increased Notch-1 signaling pathway activity, which is involved in apoptosis, in particular in the initiation phase [186]. In transgenic C57BL/6J mice, carrying a mutation for the adenomatous polyposis coli (*APC*) gene, supplementation of diet with olive oil and salmon oil (rich in omega-3 fatty acids) inhibited the cancer development. The effects of oil diet are evaluated according to the level of signal transduction and activator of transcription 3 (p-STAT3), a transcription factor involved in up-regulation of anti-apoptotic genes and the level of fatty acid synthase, a key enzyme in neoplastic lipogenesis activities. The diet rich in omega-3 fatty acids inhibited the development of the malignancy by the reduction in the levels of STAT-3 and fatty acid synthase [187].

#### 5.2.8. Dietary Fiber

Regarding dietary fiber, a polysaccharide from *Lentinus edodes* mushroom suppressed tumor growth in nude mice, upregulated caspase-3, -9 activity, increased Bax/Bcl2 ratio, increased the generation of free radicals in tumor tissues and TNF-α production [188]. Masuda Y. and co-workers demonstrated that a soluble *β-glucan* from *Grifola frondosa* inhibits tumor growth in murine cancer models through induced systemic tumor-antigen specific T cell response, increased activity of T-cells in tumor and decreased number of tumors caused immunosuppressive cells [189]. Supplementation of diet with prebiotics, such as *inulin*, in Sprague Dawley rats with azoxymethane induced CRC showed a significant increase in *Lactobacillus* sp. and *Bifidobacteria sp.* biomass along with a reduction in *E. coli* activity. In addition, the authors reported a reduction in microbial enzyme (β-glucuronidase, nitroreductase, azoreducatse) activity along with a reduction in aberrant crypt foci formation [190]. Besides, a combination of inulin with galacto-oligosaccharides significantly reduced β-glucosidase, nitroreducatse, azoreductase activity and enhanced short-chain fatty acid production [191]. Recent data reported that inulin decreased COX-2 and NF-κB expression in the colon with a significant reduction of inflammation markers [192]. Reduced inflammatory response in the jejuna and colon mucosa was also observed for co-administration of inulin and *Lactobacillus plantarum* in rats exposed to dimethylhydrazine [194].

Experiments performed with Sprague Dawley rats with 1,2 dimethylhydrazine induced CRC, showed that inulin significantly decreased the activity of colon enzymes (β-glucosidase, β-glucuronidase) and the effect was more pronounced compared to lactulose, a non-absorbable sugar [193]. Association between water-soluble polysacharide (glucomannan) and prebiotics (inulin), before initiation of CRC with azoxymethane (AOM) in a mouse model up-regulated gene expression of antioxidant enzymes, such as glutathione peroxidase 2 (GPX2), glutathione-S-transferase (GST) and catalase (CAT) and increased short fatty acid chain fatty acids production [195].

In conclusion, administration of dietary compounds to laboratory animals with induced CRC has indicated beneficial effects on different stages of carcinogenesis (Table 4). Several mechanisms are involved: Inhibition of tumor growth, modulation of several pathways (Wnt/β catenin, PI3K/Akt, Notch-1), anti-inflammatory activity, up-regulation of antioxidant enzymes and NRf2 expression, down-regulation of microbial enzymes activity, activation of caspases or increased Bax/Bcl-2 ratio.

## 6. Chemoprevention of CRC by Dietary Compounds in Humans

### 6.1. Phenolic Compounds 

#### 6.1.1. Isoflavones

Keeping in mind the important role of healthy diet (the consumption of fruits, vegetables, cereals) for CRC prevention [201], we would like to consider the role of phenolic and non-phenolic compounds in chemoprevention of CRC in humans. An epidemiological case-control study performed on Japanese patients (721 cases and 697 control subjects) between 2004–2008 revealed an inverse association between dietary isoflavone intake (as tofu or miso soup) and the risk of colorectal carcinoma in both men and women [202]. According to another case control study performed in Korea, which investigated the effects of isoflavone intake (for 901 cancer cases and 2669 controls) for CRC prevention, the highest intake of soy products and isoflavones was associated with a significantly reduced CRC risk for both men and women. The observed effects are more pronounced for rectal (in women) and distal (in men) colon cancers. A significantly reduced CRC risk was also observed for post-menopausal women [203]. Reports from a Spanish case control study (The Bellvitge CRC study) showed that exposure to isoflavones was inversely related to CRC risk. Isoflavones protect against colorectal carcinogenesis through their estrogenic properties and cause up-regulation of estrogen ERβ receptors. Moreover, isoflavones inhibit the development of CRC via up-regulation of protein p21 expression, decrease in the expression of the proliferating cell antigen (PCNA), decrease in the extracellular signal regulated kinase (ERK), Akt and nuclear factor NF-κB expression [204]. Plasma phytoestrogens were strongly linked with decreased incidence of the CRC. Analysis of plasma phytoestrogens levels in Korean and Vietnamese patients, revealed an inverse correlation between plasma genistein concentration and CRC [205]. Furthermore, genistein has shown anti-oncogenic action by increasing the expression of antioxidant enzymes [206], and by preventing CRC metastases [207].

#### 6.1.2. Lignans

According to earlier reports association between insoluble fibers, flaxseed dry extract (with 20% *secoisolariciresinol*) and milk thistle extract (with 30% silibinin, the major active constituent of sylimarin) in patients with sporadic colonic adenomas, significantly increased ERβ proteins and ERβ/ERα ratio. Moreover, increased ERβ/ERα ratio was associated with pro-aptotic effects, such as activation of caspase-3 and caspase-8 activity [208]. Another report that evaluated oral supplementation with a mixture of sylimarin and secoisolariciresinol diglucoside in patients with familial adenomatous polyposis revealed a significant reduction in the number and the size of the polyps. The observed effect was the consequence of lignans on ERβ selective agonist activity [209]. In a case control study in Korean and Vietnamese population (2003–2007), the authors did not find a correlation regarding *enterolactone* (metabolite of lignans) levels [205].

The summary of the association between CRC risk and phenolic/non-phenolic compounds is presented in Table 5.

#### 6.1.3. Anthocyanidins

Administration of a bilberry dry extract (mirtocyan), with a 36% *anthocyanin* concentration (represented by *cyanidin-3-galactoside, delphinidin-3-galactoside, delphinidin-3-arabinoside, cyanidin-3-glucoside*) in patients with CRC conducted to decreased proliferation of cancer cells and reduction of the proliferation marker Ki-67 level [212]. Recent research has shown that administration (orally as a powder or as rectal suppositories) of a black raspberry freeze-dried extract rich in anthocyanins (cyanidin-3-glucoside, cyanidin-3-sambubioside, cyaniding-3-rutinoside, cyanidin-3-xylorutinoside) to patients with familial adenomatous polyposis (FAP), significantly decreased the burden of polyps. Several mechanisms are involved: Decrease of the DNA (cytosine-5)-methyltransferase 1 (DNMT1) which was correlated with reduced methylation of p16 protein and regulatory genes in the Wnt signaling pathway [213].

### 6.2. Non-phenolic Compounds

#### 6.2.1. Organosulfur Compounds

In a double-blind placebo control study, using 51 patients, which are diagnosed with colorectal adenomas using colonoscopy, administration of aged garlic extract, especially at high dose significantly reduced the number and size of colon adenoma after 12 months [214]. Aged garlic extract is a processed garlic product, for which *allicin* is transformed into more stable compounds, such as S-allylmercaptocysteine [214]. However, other authors did not find a strong support regarding garlic chemopreventive effects, even after seven years of follow up [215]. Although, preclinical data support garlic use for CRC, a clinical study performed on both women and men for up to 24 years did not support garlic intake (even > 1 serving/week) or garlic supplementation for CRC chemoprevention [216]. Contradictory results are also reported by other authors, which even found a significant increase of colorectal incidence with 35% at a five-year follow up [217]. The contradictory effects regarding garlic chemopreventive role, are probably due to heterogeneity of studies regarding the assessment of garlic intake, type of garlic and pathology (CRC, adenoma etc). A meta-analysis of observational studies regarding the consumption of cruciferous vegetables (cabbage, broccoli) revealed an inverse correlation between dietary intake and CRC risk [236].

#### 6.2.2. Carotenoids

A large European case control study showed an inverse correlation between dietary concentration of *β-carotene*, vitamin C and CRC incidence, mainly in the distal colon [219]. Kabat C. G. and co-workers have also found an inverse correlation between *β-carotene* plasma levels and CRC incidence in post-menopausal women [220].

#### 6.2.3. Polyunsaturated Fatty Acids

In a phase II double-blind randomized clinical trial for patients with CRC and liver metastasis, pre-operative administration of *EPA* for a median of 30 days conducted to increased levels of EPA and decreased level of PGE2 in tumor tissues compared to control tissue. Moreover, EPA also showed anti-angiogenic activity, whilst preoperative treatment with omega-3 fatty acids had postoperative benefit, in the first 18 months (regarding cancer recurrence). This effect may be the consequence of EPA prolonged plasma biological half-life [221]. According to a prospective study is US men and women, intake of marine omega-3 fatty acids and fish are inversely associated with rectal cancer incidence [222]. In a Japanese based prospective study, administration of omega-3 fatty acids reduced the incidence of proximal colon cancer [223].

According to earlier studies, administration of encapsulated fish oil to patients with CRC, undergoing chemotherapy, significantly reduced C reactive protein (CRP) and CRC/albumin ratio compared to controls. Increased values of CRP and CRP/albumin ratio are associated with a chronic inflammatory state and poor clinical prognosis for CRC patients. Administration of fish oil also increased EPA, DHA and decreased arachidonic acid plasma levels [224]. It is well known that polyunsaturated fatty acids have anti-inflammatory effects; a meta-analysis of clinical trials with CRC patients (that included administration of fish oil or supplementation of diet with omega-3 fatty acids) showed a significant decrease of IL-6 plasma levels and an increase for albumin concentration. For patients undergoing chemotherapy supplementation with EPA and DHA significantly reduced CRP/albumin ratio [225]. Other authors reported that administration of an enriched omega-3 nutritional supplement significantly increased EPA, DHA and decreased arachidonic acid concentration in granulocytes. These findings suggest that omega 3 fatty acids might exert an immuno-stimulatory effect [226].

However, other studies failed to demonstrate a beneficial role of omega-3 fatty acids for CRC patients. According to Ma C.J. and coworkers, administration of a lipid emulsion composed of soybean oil, medium chain triglycerides and polyunsaturated fatty acids after surgery of CRC patients did not improve the level of the inflammatory markers, instead had a positive effect on lipid profile [227]. In addition, subjects with a high intake of fish and marine food appeared to have an increased risk of distal colon cancer. These differences might be the consequence of high pH values in the distal colon (due to low production of short chain fatty acids), that could attenuate omega-3 fatty acids effects or the presence of contaminants in fish (lead, mercury etc.) [222].

#### 6.2.4. Dietary Fiber

Recent research has shown that supplementation with agave *inulin* in healthy patients has a beneficial role upon gut microbiota. Administration of the agave inulin resulted in significant increase of *Bifidobacterium* genus. A significant decrease was observed for *Rumincoccus sp*. and *Desulfovibrio sp.*, which might have a benefit towards CRC incidence [228]. Still other authors did not find a correlation between inulin intake and CRC risk. In a randomized phase II chemoprevention trial, with patients with aberrant crypt foci (ACF) the administration of inulin did not show a significant reduction in ACF number compared to control [229].

A high fibers intake is associated with low risk of CRC. In a prospective cohort study, intake of cereal-derived fibers was associated with a low risk of *Fusobacterium nucleatum* positive CRC. Recent findings have shown that a high amount of *F. nucleatum in* tumor tissues was linked to cancer severity and high mortality [230]. A meta-analysis of studies regarding the benefit of dietary fiber consumption, have also shown an inverse correlation between fiber intake and risk of colorectal adenoma [231,232]. According to Kunzmann A. and co-workers a diet rich in fibers significantly reduced CRC risk. Nonetheless, the association was stronger for males than females [233]. In another multicenter prospective cohort study, a high intake of dietary fibers was significantly associated with low risk of distal/proximal colon and rectum cancers [234]. Navarro S. and co-workers investigated the effect of dietary fiber and omega-3, -6 fatty acids in a women’s health initiative prospective cohort during their 11.7-year follow up. The results pointed out a reduced incidence of CRC for the association between a low dose of soluble fiber, a high dose of insoluble fiber and a high dose of EPA and DHA [237]. However, results from other studies regarding the benefit role of dietary fiber for CRC prevention showed contradictory results. In a randomized control trial, administration of resistant starch, which exerts similar effects as dietary fiber in patients with Lynch syndrome, a hereditary non-polyposis colon cancer, over 29 months, had no detectable effect on cancer development [235].

In conclusion, most of the presented clinical trials have shown an inverse correlation between dietary intake of carotenoids, phytoestrogens (lignans, isoflavones), polyunsaturated fatty acids, fibers and CRC incidence (Table 5). However, results should be carefully interpreted keeping in mind the individual variability, the number of dietary compounds consumed by patients and the complexity of CRC.

## 7. Bioavailability of the Natural Dietary Compounds

Regularly, when discussing the health value of the dietary compounds, these are evaluated according to their ability to be released from the ingested food, be absorbed in the gastrointestinal tract, undergo the metabolic steps and reach the target tissue. In the case of CRC, the last steps are overcome, since the interaction between dietary compounds and cancer cells is more direct. At the intestinal lumen, the lipophilic compounds can pass into enterocytes by facilitated diffusion, while the hydrophilic compounds (such as polyphenols) can enter the cells after the aglycone is liberated through the enzymatic hydrolysis [238]. However, the bioavailability can be reduced by the presence of ABC transporters (ATP-Binding Cassette) or other multidrug mechanisms, which increase the efflux of xenobiotic [3]. In CRC cells this effect might be reversed by the administration of the flavonoids from *Citrus sp.* plants [239]. With the aim of understanding the beneficial properties of the dietary compounds, their bioavailability is required to be studied (Table 6).

The plasma concentration of the dietary compounds is another key topic to discuss. Although experiments performed in cancer cells are important for developing new therapeutic agents, differences occur when the same substance is administrated in animal models or used in clinical trials due to its bioavailability, metabolisms or interaction with gut microbiota. A series of extensive reviews presented by Manach et al., regarding the bioavailability of the polyphenols, introduced the average values of the polyphenols in plasma at a short time after ingestion [240]. The concentration values of the plasma polyphenols vary in range of low micromolar levels. For instance, after the ingestion of ~100 mg quercetin from apples the plasma level of quercetin reached 0.3 μM at 2.5 h after consumption, while ingestion of ~125 mg hesperitine from orange juice reached 2.2 μM at 5.4 h after consumption. Similar plasma concentration levels are identified for epigallocatechin-3-*O*-gallate (EGCG) from green tee, namely 0.16–0.96 μM after ingestion of 200–800 mg EGCG or genistein from soymilk, i.e., 1.14 μM subsequently to the consumption of 0.59 mg/kg [49,72,240,241]. Several strategies, such as modulation of the administration, inhibition of the metabolic pathways, administration in combination with other drugs or encapsulation methods attempts to improve the bioavailability of the dietary compounds [120,238]. The bioavailability of the phenolic compounds was extensive studied [120,238,242,243,244], while little is known about the bioavailability of dietary non-phenolic products. The maximum concentration of ellagitannis in 1 h after the intake of 318 mg ellagitannins from pomegranate juice was about 0.06 μM [245]. The non-alkaloid capsacin administrated to in rats (30 mg/kg body weight) was detected in serum after 1 h in ranges of 1.9 ± 1.2 μM and the concentration levels decreased within time [246]. An estimation of 50 g of olive oil/day uptake leads to the detection of ~13 μM hydroxytyrosol (a metabolite of oleuropein obtained after the hydrolysis of oleuropein-aglycone) in plasma, a concentration much lower than 50–100 μM required for the antioxidant effect. The authors concluded that the olive oil compounds are well absorbed, but the plasma level of their metabolites are too low to induce substantial biological effects [247]. Further research in this field is needed to analyze and improve the bioavailability of non-phenolic compounds in order to obtain significant effects on human health.

### 7.1. Bioavailability of Phenolic Compounds

Studies demonstrated the small bioavailability of *curcumin* was due to poor absorption, rapid metabolism and rapid elimination. In order to increase curcumin bioavailability it was associated with other compounds, for instance piperine, the main component of black pepper, which increased its bioavailability by 2000% [248]. Another method, by which it has been demonstrated to obtain a better bioavailability of curcumin, was the method of using nanocurcumin, a polymeric nanoparticle encapsulated formulation of curcumin. Nanocurcumin has shown a better activity in cancer cell lines by inhibition of NF-κB [259]. There are studies in vivo made in both humans and animals, to determine the bioavailability of curcumin. Experiments on rats showed a maximum serum concentration (*C_max_*) of 1.35 ± 0.23 μg/mL in 0.83 h compared to humans where *C_max_* in the same period was much lower consisting in 0.006 ± 0.005 μg/mL [120].

Studies made with *EGCG* both in vivo and in vitro are conducted to show the systemic absorption of the most abundant catechin found in the green tea. Derliz Mereles and Werner Hunstein proved that for optimizing EGCG bioavailability there must be taken into consideration the pharmacokinetic parameters that can diminish or enhance the bioavailability. Thereby among the factors that improve the absorption of EGCG are alkaloids like piperine, vitamin C, proteins like albumin or even fish oil. On the other hand, the absorption of EGCG can be reduced by air contact oxidation, metal ions like Ca^2+^ and Mg^2+^, temperature or beverages like milk. In vivo studies conducted on human subjects, after administration of one oral dose of EGCG with a fasting period overnight, *C_max_* was reached in 1–2 h and the elimination half-life of EGCG was at 3.4 ± 0.3 h [249]. Thereby the matter of bioavailability is a major concern for all the scientists who are trying to find a better way for natural compounds to be assimilated and to keep their effects powerful.

Beside curcumin and EGCG, the natural compound *resveratrol* is also considered to have a poor solubility and bioavailability [260]. Thus, studies on resveratrol showed that a better bioavailability can be achieved by combining resveratrol with other foods, beverages or even with other polyphenols. Calvo-Castro, L. A. et al. demonstrated that the impact of trans-resveratrol, on a group of six men and women, was increased by a liquid micellar formulation of a grapevine-shoot extract, which contains a high amount of resveratrol monomers and oligomers [250]. The experiment demonstrated that a single dose of 500 mg of grapevine-shoot extract (30 mg trans-resveratrol, 75 mg trans-ε-viniferin) influenced the trans-resveratrol concentrations with a *C_max_* by 10.6-fold higher with no detection of trans-ε-viniferin nor in plasma or urine [250].

*Quercetin* a flavonoid recognized for the powerful effect in cancer cell lines was also studied for its bioavailability. According to Khan, F. et al. a study conducted on rodents demonstrated that after an administration of quercetin intravenously, after plasma assays are performed, no quercetin was found [261]. These results might be explained by the fact that is difficult to assess the intracellular effect of the compound at the organ site and nowadays is generally accepted the correlation between the plasma concentration of the compound and its therapeutic action [262]. A high quantity of quercetin is found in onion, which is considered to be more effective than quercetin supplement regarding its bioavailability, presumably for the increased intestinal absorption of the food matrix [263]. It has been demonstrated that the absorption of quercetin might depend on the type of sugar residue attached to quercetin. For instance, quercetin glicosides found in onion are better absorbed unlike the major quercetin glycoside in tea. The assessment of quercetin glucoside absorption was between 3%−17% in healthy subjects, after a dose of 100 mg which is relatively low due to poor absorption, extensive metabolism or rapid elimination [251].

Another flavonoid, worth to be taken into consideration is *genistein* found in soy and is well known for its beneficial properties, including multiple molecular effects, such as the promotion of apoptosis, anti-inflammatory properties, modulation of metabolic pathways and steroidal hormone receptors [264]. For a better understanding of how genistein can work as a chemopreventive agent, it is required to know its bioavailability. Pharmacokinetic studies demonstrated the low oral bioavailability of genistein, whilst the plasma or tissue concentrations are reduced compared to in vitro values of half maximal inhibitory concentration, IC_50_. Yang. Z. et al. demonstrated in their study that after intravenous and oral administration of 20 mg/kg genistein in FVB mice, genistein was transformed mostly to glucuronosides and sulfates and the genistein aglycone bioavailability was 23.4% [252]. Regarding the oral bioavailability of genistein there are studies that showed a better bioavailability of total genistein in mice than genistein aglycone. After feeding female Balb/c mice with soy protein, Yang Z. et al. demonstrated a difference between bioavailability of total genistein, which was almost 90%, and genistein aglycone, less than 15% [252]. In addition, it should be taken into account supplementary factors which can contribute to poor absorption of genistein, such as age, gender or dose frequency, but the most important factor is related to absorption, distribution, metabolism and excretion (ADME) properties [252].

*Anthocyanins* are a group of molecules that belong to flavonoid family and they are found in a large group of fruits, flowers, roots and leaves responsible for the blue, purple and red color [265]. In contradiction to other flavonoids, the bioavailability of anthocyanins (delphinidin, malvidin, cyaniding and pelargonidin) is rather different. These molecules can be absorbed in the stomach or intestines. In the gastroinstestinal wall, it may be found the pure form of cyanidin-3-glucoside anthocyanin and pelargonidin-3-glucoside, which can be decomposed by microbiota right after reaching to the large intestine [265]. In a pilot study, Muller D. et al. demonstrated that anthocyanins could reach the small intestine within one hour and the level of the compound at the intestinal area was about 30–50% of the ingested substance, while the plasma level was very low [253]. Anthocyanins can be methylated due to the metabolic transformation carried out by the enzymes, such as cathechol-*O-*methyltransferase (COMT), thereby the anticancer effect of anthocyanins may be limited. However, there are inhibitors that are able to decrease the methylation of polyphenols [266].

*Proanthocyanidins* are phytochemicals represented by a group of flavonoids which are found in a variety of plants and aliments, such as apples, cinnamon, grapes, cranberry, green tea etc. [267]. However, the main downside of proanthocyanidins is regarding its bioavailability. Multiple studies are conducted to elucidate the bioavailability issue of this class of oligomeric flavonoids. While the monomeric flavonoids are rather absorbed in the small intestine, the metabolism of oligomeric and polymeric proanthocyanidins in the colon, is not so much understood. In that matter Choy Y. et al. conducted a study to investigate the presence of proanthocyanidins in the colon after ingestion of grape seed extract [254]. The evidence from this study suggests the presence of proanthocyanidins in the colon as intact compounds which might be beneficial for maintaining a healthy digestive system [254].

### 7.2. Bioavailability of Non-Phenolic Compounds

*Capsaicin* is one of the most pungent ingredients consumed worldwide and is naturally found in chili peppers. Capsaicin is known to possess many beneficial effects on the human body, such as anti-inflammatory, antimicrobial, anticancer and it was described to work as a topical analgesic. Beside capsaicin, capsaicinoids are also represented by dihydrocapsaicin (DHC), nordihydrocapsaicin (n-DHC), homocapsaicin (h-C) and homodihydrocapsaicin (h-DHC). However, capsaicin low bioavailability is a concern for restricting its application [268]. Rollysons, W.D. et al. conducted a study in vivo to explore the intestinal absorption of capsaicin, using lab rats to isolate intestinal sacs. The capsaicin was absorbed into intestinal tissues, jejunum and serosa fluid [255]. For instance, the absorption of the compound was differently regarding the intestinal region of interest. Accordingly, 1 mM of capsaicin was absorbed in a proportion of 50% in the stomach, 80% in the jejunum and 70% in the ileum [255]. Kawada et al. conducted a study on WISTAR rats are the effect of capsaicin and DHC are absorbed in the stomach and small intestine in a percentage of 85% [246].

*Piperine* belongs to *Piperaceae* family and is one of the most important alkaloids found in black pepper (*Piper nigrum*) and in long pepper (*Piperum longum*). Because of its anticancer effect, it is mandatory to have knowledge about the potential of absorption and the bioavailability of piperine. It is well known that piperine is insoluble in water and presents a low bioavailability, which may limit its use in clinical experiments. Nevertheless, piperine can be used in clinical assays single or in addition to other dietary agents. Several studies demonstrated the ability of piperine to act as a bioavailability enhancer for many chemopreventive agents, such as resveratrol, leading to increased levels of revesterol in plasma [269]. Johnson J. J et al. demonstrated that the administration of resveratrol alone or in combination with piperine in vivo, enhanced the serum bioavailability of resveratrol by almost 229% [256]. In addition, piperine was known to enhance the effect of curcumin and lycopene because of its ability to inhibit intestinal and hepatic glucoronidation. For instance, piperine enhanced the bioavailability of curcumin by 2000%, in both rats and human experiments [257].

*Allicin* is derived from isothiocyanate phytochemical and is a compound found in garlic. Among its properties are included antifungal, anti-neoplastic and antibacterian effects [270]. However, these properties are questionable because of the poor bioavailability of allicin. According to Lawson et al. after oral administration of garlic and pure allicin, there was no detection of it neither in urine nor in blood [258].

In conclusion, the oral bioavailability of dietary agents was variable depending on each compound of interest, being able to be increased or decreased by other agents, vitamins, proteins according to each experiment. Further studies should be carried out to have an objective vision regarding the ability of the natural compounds to help in chemoprevention.

### 7.3. Encapsulation Strategies for Increased Bioavailability

Since the absorption of the dietary compounds, after their oral administration, might by restricted by the insufficient gastric residence time, low permeability, low solubility, instability during food processing (pH, enzymes, presence of other nutrients) the health benefits of these compounds are limited. During the last decades the nanoparticles are investigated due to their ability to transport and deliver drugs. Several advantages might recommend the nanoparticles as potent delivery agents: Increased bioavailability, reduced toxicity and improved solubility in aqueous medium [271]. Furthermore, the nanoparticle can accumulate in the solid tumors as a result of the reduced lymphatic drainage [272]. In this view, many anticancer drug delivery systems are developed based on nanoscale strategy, by using nanoparticles of different compositions [273]. “Yallapu et al. demonstrated that curcumin loaded PLGA nanoparticles display an increased effect on metastatic cancer cells than curcumin alone [274], while Radu et al. developed a poly alkanoate nanocarrier for silymarin delivery with good drug release and biocompatibility properties [275]. Despite its extensive in vitro study, the drugs encapsulation approach is still under development and only a few clinical trials are currently running [276].

The most common carrier agents used in micro- and/or nano-encapsulation of dietary bioactive compounds are (i) polysaccharides, such as: Starch [277], dextrins [278], maltodextrins [279], cyclodextrins [280], (ii) celulloses, such as: Carboxymethyl cellulose, methylcellulose [281] and cellulose ethers [282], (iii) pectins [283], (iv) chitosan [284] etc. To obtain these micro- and/or nano-encapsulations several techniques are developed, of which spray drying, freeze drying, complex coacervation, emulsification, anti-solvent precipitation, extrusion, electro-spinning, layer-by-layer deposition and solid dispersion are the most frequently used.

## 8. Conclusions

Natural compounds can exert anti-tumor effects due to their antioxidant capacity and their ability to inhibit cellular growth, capacity to trigger tumor cells apoptosis or to modulate the metastatic cascade. The use of natural bioactive compounds could minimize chemotherapy and/or radiotherapy side effects, such as neutropenia, diarrhea, cardiotoxicity, nephrotoxicity, hepatotoxicity, etc.

Both in vitro and in vivo studies demonstrated that the administration of dietary active compounds induces growth inhibition, apoptosis and inhibition of adhesion and migration. Furthermore, they exert anti-inflammatory effects and modulate Wnt/β catenin, PI3K/Akt and Notch-1 key pathways for tumor initiation.

Surprisingly, some clinical trials presented in this review have shown an inverse correlation between dietary intake of carotenoids, phytoestrogens (lignans, isoflavones), polyunsaturated fatty acids, fibers and CRC incidence, but these results should be carefully interpreted considering the individual variability, co-morbidities and cancer development status. Consequently, further investigations should focus on underlying dietary compounds efficacy and the safety use in combination with chemotherapy and/or radiotherapy, as well as their bioavailability (intestinal absorption and metabolism).

With this respect, the oral bioavailability of the dietary agents was shown to be variable depending on each compound and to be influenced by other agents. However, most of the studies conclude that generally, the dietary bioactive compounds show low bioavailability. Therefore, the development of new strategies to increase their bioavailability and adjust their administration doses would be of great interest. A promising approach in this view is the development of nano-/micro-shuttles able to carry active bio-compounds and to release these molecules in a controlled manner.

## Figures and Tables

**Table 1:** Main classes of phenolic compounds with representative members, chemical structure and dietary sources [58,72,73,74].

Dietary Compounds	Chemical Structure	Representative Compounds	Sources
**Flavonoids**			
**Flavones**	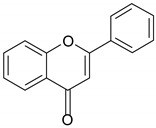	Apigenin, luteolin	Celery (*Apium graveolens* L.), onions (*Allium cepa* L.), broccoli (*Brassica oleracea italic* Plenck.), red orange (*Citrus x sinensis* L. Osbeck), green pepper (*Capsicum sp*.)
**Flavonols**	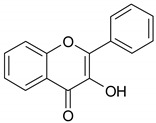	Quercetin, kaempferol, myricetin	Broccoli (*Brassica oleracea italic* Plenck.), lettuce (*Lactiva sativa* L.), onions (*Allium cepa* L.), kale (*Brassica oleracea* L.), leek (*Allium ampeloprasum* L.), apricot (*Prunus armeniaca* L.), apple (*Malus domestica* Borkh.), blueberry (*Vaccinium corymbosum* Rydb.), black currant (*Ribes nigrum* L.)
**Flavonones**	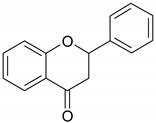	Naringenin, hesperitin, diosmetin	Orange (*Citrus x aurantium sp.* L.), grepfruit (*Citrus paradise* Macfad.), lemon (*Citrus limon* L.)
**Isoflavones**	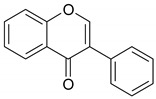	Genistein, daidzein, glycitein	Soybeans (*Glycine max* L.) boiled, miso, tofu, soy milk, soy flour
**Flavan-3-Ol** Monomeric catechins	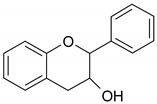	Catechin, epicatechin, epigallocatechin, epigallocatechin-3-O-gallate	Apple (*Malus domestica* Borkh.), peach (*Prunus persica* L.), green tea, black tea (*Camelia sinensis* L.), red wine, grapes (*Vitis vinifera* L.), beans (*Phaseolus vulgaris* L.)
Protoanthocyanidins or condensed tannins	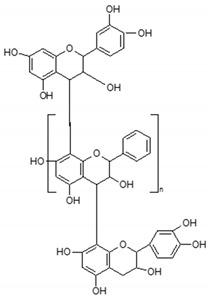 Protoanthocyanidin B	Proanthocyanidin A, B	American cranberry (*Vaccinium macrocarpon* Aiton.), cranberry (*Vaccinium oxycoccus* L.), chokeberries (*Aronia melanocarpa* Elliott.), plum (*Prunus domestica* L.), blueberry (*Vaccinium myrtillus* L.), red currant (*Ribes rubrum* L.), lingonberries (*Vaccinium vitis-idaea* L.), pear (*Pyrus communis* L.), apple (*Malus domestica* Borkh.), almonds (*Prunus dulcis* Mill.), hazelnuts (*Corylus* spp.), pecans (*Carya illinoinensis* Wangenh.), pistachio nuts (*Pistacia vera* L.), walnuts (*Juglans regia* L.), grape seeds (*Vitis vinifera* L.), beans (*Phaseolus vulgaris* L.), cowpea (*Vigna unguiculata* Walp.), lentils (*Lens culinaris* Medik.), barley (*Hordeum vulgare* L.), rice (*Oryza sativa* L.), sorghum (*Sorghum bicolor* L. Moench.)
**Anthocyanidins/Anthocianins**	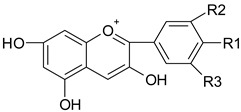	Delphinidin (R_1_, OH; R_2_, OH; R_3_, OH); cyanidin (R_1_, OH; R_2_, OH; R_3_, H) malvidin (R_1_, OH; R_2_, OCH_3_; R_3_, OCH_3_); pelargonidin (R_1_, OH; R_2_, H; R_3_, H)	Blackberry (*Rubus fruticosus* L.), raspberry (*Rubus idaeus* L.), bilberry (*Vaccinium myrtillus* L.), plum (*Prunus sp*), sour cherry (*Prunus cerasus* L.), black grapes (*Vitis vinifera* L.), elderberry (*Sambucus nigra* L.), red cabbage (*Brassica oleracea* L.)
**Non-Flavonoids**			
**Tannins** A. Gallotannins derivatives	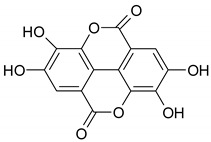	Ellagic acid	Raspberry (*Rubus idaeus* L.), strawberry (*Fragaria x ananassa* Duchesne.), pomegranate (*Punica granatum* L.), black currants (*Ribes nigrum* L.), blackberry (*Rubus fruticosus* L.), guava (*Psidium guajava* L.)
B. Elagotannins derivatives	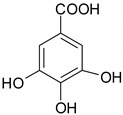	Gallic acid	
**Phenol-Carboxilic Acids** A. Hydroxybenzoic acids	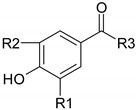	Gallic acid (R_1_, OH; R_2_, OH; R_3_, OH), syringic acid (R_1_, OCH_3_, R_2_, OCH_3_, R_3_, OH)	Broccoli (*Brassica oleracea italica* Plenck.), cowpea (*Vigna unguiculata* Walp.), spinach (*Spinacia oleracea* L.), black currant (*Ribes nigrum* L.), acai palm (*Euterpe oleracea* Mart.), sugar apple (*Ananona squamosa* L.)
B. Hydroxy-cinnamic acids	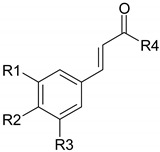	Cinnamic acid (R_1_, H; R_2_, H; R_3_, H; R_4_, OH), Caffeic acid (R_1_, OH; R_2_, OH; R_3_, H; R_4_, OH), chlorogenic acid, ferulic acid acid (R_1_, H; R_2_, OH; R_3_, OCH_3_; R_4_, OH), sinapic acid (R_1_, OCH_3_; R_2_, OH; R_3_, OCH_3_; R_4_, OH), *p*-coumaric acid (R_1_, H; R_2_, OH; R_3_, H; R_4_, OH), rosmarinic acid	Pear (*Pyrus communis* L.), kiwi (*Actinidia deliciosa* C.F.Liang and A.R.Ferguson), plum (*Prunus domestica* L.), apple (*Malus domestica* Borkh.), artichoke (*Cynara scolymus* L.), potato (*Solanum tuberosum* L.), coffee (*Coffea arabica* L.), rosemary (*Rosmarinus officinalis* L.), basil (*Ocimum basilicum* L.), oregano (*Origanum vulgare* L.), sage (*Salvia officinalis* L.)
**Lignans**	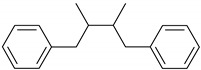	Secoisolariciresinol, sesaminol, sesamol, sesamin	Flaxseed/ linseed (*Linum usitassimum* L.), sesameseed (*Sesamum indicum* L.)
**Stilbens**	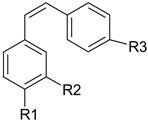	Resveratrol (R_1_, OH; R_2_, OH; R_3_, OH)	Red wine, red grapes (*Vitis vinifera* L.), plum (*Prunus domestica* L.)
**Other Compounds**	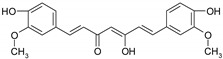	Curcumin	Turmeri*c* (*Curcuma longa* L.)
	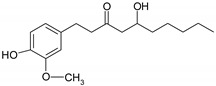	Gingerol	Ginger (*Zingiberis officinale* Roscoe.)

**Table 2:** Main classes of dietary compounds (others then phenolic compounds) with representative members, chemical structure and dietary sources [52,55,62,66,72,73,75,76,77,78,79].

Dietary Compounds	Chemical Structure	Representative Compounds	Sources
**Carotenoids**	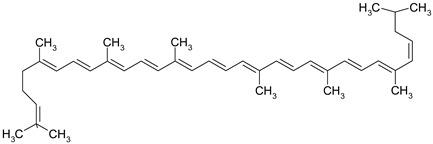	Lycopene	Tomatoes (*Solanum lycopersicum* L.), guava (*Psidium guajava* L.), pumpkin (*Cucurbita pepo* L.), carrot (*Daucus carota subsp. sativus*), rose hip (*Rosa rugosa* Thunb.), watermelon (*Citrullus lanatu*s Thunb.), saffron (*Crocus sativa* L.)
	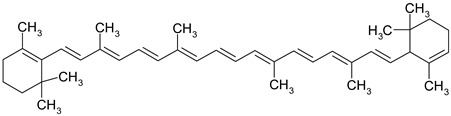	α-carotene	
	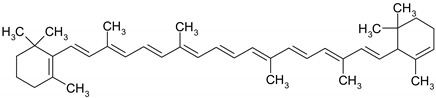	β-Carotene	
	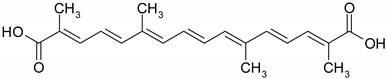	Crocetin	
**Iridoids**	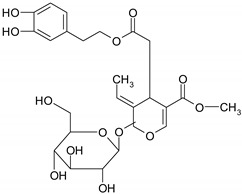	Oleuropein	Unripe olive fruits, table olives, virgin olive oil (*Olea europaea* L.)
**Nitrogen Compounds** A. Alkaloids	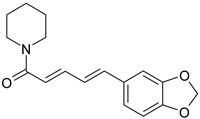	Piperine	Black pepper (*Piper nigrum* L.); long pepper (*Piper longum* L.)
B. Non-alkaloidic compounds	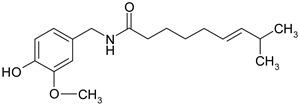	Capsaicin	Chilli pepper (*Capsicum* sp.)
**Organosulfur Compounds**	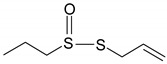	Allicin	Garlic (*Allium sativum* L.), onion (*Allium cepa* L.), broccoli (*Brassica oleracea italica* Plenck.)
	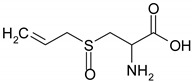	Allin	
	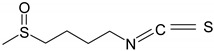	Sulforaphane	
**Phytosterols**	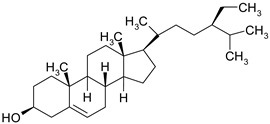	β-sitosterol	Zucchini (*Cucurbita pepo* L.), lettuce *(Lactuca sativa* L.), mangold (*Betta vulgaris* L.), white cabbage (*Brassica oleracea* L.), pumpkin seed (*Cucurbita pepo* L.), oat (*Avena sativa* L.), peanut (*Arachys hypogaea* L.), mushrooms (*Agaricus bisporus, Lentinula edodes, Grifolia frondosa, Boletus edulis, Pleurotus osteatrus, Armillaria mellea*)
	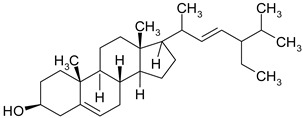	Stigmasterol	
	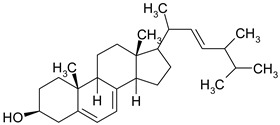	Ergosterol	
	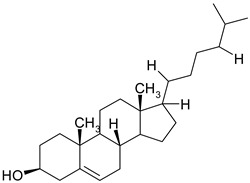	Campesterol	
**Essential Oil Compounds**	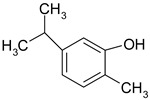	Thymol	Essential oils of thyme (*Thymus vulgaris* L.), wild thyme (*Thymus serphyllum* L.), oregano (*Origanum vulgare* L.), lemon balm (*Melissa officinalis* L.), cinnamon (*Cinnamomum* sp.), aniseed (*Pimpinella anisum* L.), star anise (*Illicium verum* Hook.), fennel (*Foeniculum vulgare* Mill.), black cumin (*Nigella sativa* L.)
	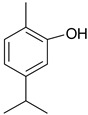	Carvacrol	
	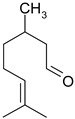	Citronellal	
	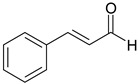	Cinnamaldehyde	
	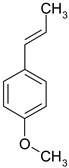	Anethole	
	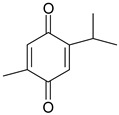	Thymoquinone	
**Polyunsaturated Fatty Acids** A. Omega-3 fatty acids	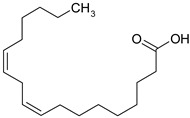	α-linolenic acid (ALA)	Catfish (*Silurus glanis*), anchovy (*Engraulis encrasicolus*), bluefish (*Pomatomus saltatrix*), sardine (*Sardina pilchardus*), tuna (*Thunnus thynnus*), spiny lobster (*Panulirus argus*), mussels (*Mytilus edulis*), oyster (*Ostrea lurida*), herring (*Clupea harengus membras*, *C. h. harengus*), mullet (*Mugil cephalus*), shrimps (*Palaemon serratus*), seaweeds (*Rhodophyta phylum*, *Phaeophyceae* class, *Chlorophyta phylum*); seed oil from: Hemp (*Cannabis sativa* L.), chia (*Salvia hispanica* L.), echium (*Echium plantagineum* L.), flax (*Linum usitassinum* L.); walnut oil (*Juglans regia* L.)
	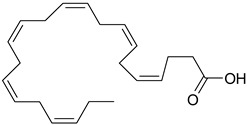	Docosahexaenoic acid (DHA)	
	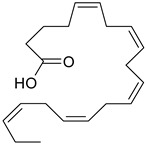	Eicosapentaenoic acid (EPA)	
B. Omega-6 fatty acids	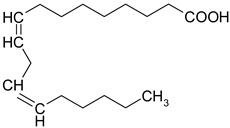	Linoleic acid (LA)	Black currant (*Ribes nigrum* L.) seed oil, gooseberries (*Ribes grossularia* L.) seed oil, hemp (*Cannabis sativa* L.) seed oil; meet from beef (*Bos taurus*), lamb (*Ovis aries*), pork (*Sus scrofa domesticus*)
	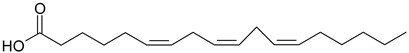	γ–linolenic acid (GLA)	
**Dietary Fiber**	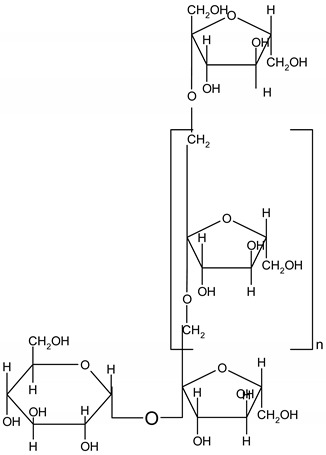	Inulin	Sweet potato (*Ipomoea batatas* L.), leek (*Allium ampeloprasum* L.), garlic (*Allium sativum* L.), onion (*Allium cepa* L.)
	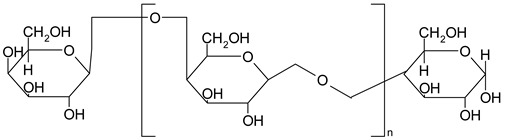	Galactooligosaccharides	Apple (*Malus domestica* Borkh.), pear (*Pyrus communis* L.), potato (*Solanum tuberosum* L.), beans (*Phaseolus vulgaris* L.), brusselsprouts (*Brassica oleracea var. gemmifera* L.), whole wheat (*Triticum* L.), whole corn (*Zea mays* L.), chickpea (*Cicer arientinum* L.), apricot (*Prunus armeaniaca* L.)
	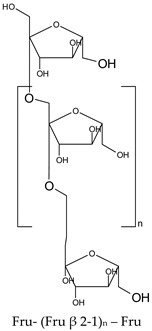	Fructooligosaccharides	
	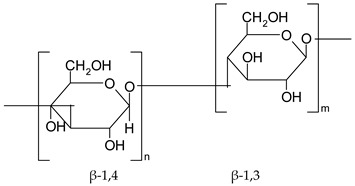	β-glucans	Mushrooms (*Agaricus bisporus, Lentinula edodes, Grifolia frondosa, Boletus edulis, Pleurotus osteatrus, Armillaria mellea*)
	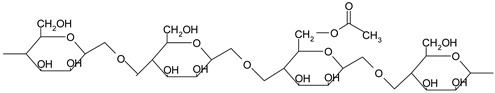	Glucomannans	Konjac (*Amorphophallus konjac* Koch.)

**Table 3:** Summary of in vivo experiments regarding CRC and phenolic compounds.

Author, Year	Animal Models	Doses and Duration of Administration	Results
**Genistein**			
Song S. et al., 2018 [157]	Mice with AOM/DSS CRC	225 mg/kg diet; 450 mg/kg diet and 900 mg/kg diet—six month	Significant improvement of colon architectural repair, anti-inflammatory activity ↓ COX-2, TNF-α; ↓ expression of PI3K/AKT pathway; ↑expression of FOXO_3,_ Bax proteins
Zhang Y. et al., 2013 [101]	Sprague–Dawley rats with AOM induced CRC	Pre-treatment diet supplementation with 140 mg/kg—six weeks, before induction of cancer	Inhibition of aberrant crypt foci, prevention of nuclear β-catenin accumulation, suppression of cyclin D1, c-myc expression and Wnt signaling genes (Wnt1, Wnt5a, Sfrp1, Sfrp5)
Son T.G. et al., 2013 [158]	BALB/mice subcutaneously injected with CT26 mouse colon cancer cells	Tumor bearing mice are treated with genistein 200 mg/kg 1 day before radiation (5, 10 Gy); evaluation of tumors after 12 h, 3.5 days	genistein increased progenitor cell survival and cell death after radiation, recovery of intestinal damage after radiation (↑ Ki-67), significant tumor regression for combined treatment
**Cyanidin/Pelargonidin/Malvidin**			
Kang S.Y. et al., 2003 [159]	Apc^min^ mice-mutant mouse lineage predisposed to multiple intestinal neoplasia due to mutations in adeno-matous polyposis coli (APC) gene	800 mg/L anthocyanidins/200 mg/L (rich in cyanidin glucosides) in the drinking water and modified diet with 200 g/kg freeze dried cherries	Fewer and smaller adenomas in the cecum compared to control Colon tumor volume was not significantly reduced
Shi N. et al., 2015 [160]	Male CRJ:CD-1 (ICR) mice with CRC induced with AOM/DSS	2.5%; 5%; 10% freeze-dried strawberries—cyanidine glucoside (1.67%) and pelargonidin glucoside (41.1%)—20 weeks	Inhibition of tumor development from 100% (control) to 74–44%, ↓ of nitrotyrosine production, ↓ Nf-kb, PI3K/AKT phosphorylation, ↓ COX-2, iNOS expression
Silva R.M. et al., 2015 [161]	Wistar rats with AOM induced CRC	Administration of 1% (222 mg/zi) or 2% red grape juice (444 mg/zi), two weeks before AOM or 4 weeks after the last administration of AOM	↓ COX-2 mRNA with 1% grape juice before AOM and with 2% juice after the last AOM administration
Fernandez J. et al., 2018 [162]	Male Fischer 344 rats with AOM/DSS induced CRC	20 g/day/rat of functional sausage with 0.11 anthocyanins (mixture of 1:1 dehydrated strawberries and blackberries powder with 59% cyanidin-3-glucosides and 41% pelargonidin-3-glucosides)	Significant reduction of Peyer patches, caecum weight, number of polyps; Significant reduction of *Bilophila wadsworthia*—a bacteria that generated high level of H_2_S and has pro-inflammatory effects
**Chlorogenic Acid**			
Matsunaga K. et al., 2002 [163]	Male F344 rats with CRC induced with AOM	250 ppm chlorogenic acid was administered one week before and a one week after tumor induction with AOM; study duration 36 weeks	Significant decrease of colon tumors for pre- treatment with chlorogenic acid
Banerjee N. et al., 2016 [164]	Sprague Dawly rats with AOM induced CRC	Plum (*Prunus salicina* L.) beverage rich in chlorogenic and neochlorogenic acids, 10 weeks	Significant decrease of dysplastic polyps, ↓ expression of COX-2, Nf-kB, AKT/mTOR signaling pathway, ↑ miR-143
**Cinnamic Acid**			
Zhu B. et al., 2016 [165]	Female BALB/c nude mice inoculated with HT29 colon carcinoma cells	1 and 1.5 mmol/kg x3/week for two weeks	Significant inhibition of tumor growth, ↑ expression of Bax and caspase 3
***p*-Coumaric Acid**			
Sharma S.H. et al., 2017 [166]	Male albino rats with DMH induced CRC	50 mg/kg, 100 mg/kg, 200 mg/kg; 15 weeks	Significant dose-dependent reduction of polyps incidence and formation of pre-neoplasic lesions, reduction of oxidative stress; significant decrease in gut microbial enzymes (mucinases and β-dehydrogenases)
**Rosmarinic Acid (RA)**			
Venkatachalam K. et al., 2013 [167]	Male wistar rats with DMH induced CRC	5 mg/kg received during administration of DMH (15 weeks) or one week after the last DMH dose (until 30 weeks) or through the whole period (30 weeks)	Supplementation with RA for the whole period showed the highest tumor reduction, ↓ stress oxidative markers, ↓mucosal bacterial enzymes activity, regulation of xenobiotic metabolizing enzymes, up-regulation of apoptotic factors
**Gallic Acid**			
Giftson J.S. et al., 2011 [168]	Male albino Wistar rats with DMH induced CRC	50 mg/kg received one week before DMH and continued 30 weeks (group 1), after cessation of DMH until 30 weeks (group 2), along the whole period (group 3)	Supplementation with gallic acid for the whole period showed the highest tumor reduction, regulation xenobiotic metabolizing enzymes, decreased tumor incidence
**Secoisoscilaresinol**			
Shah N.R. and Patel B.M., 2016 [169]	Diabetic male Sprague Dawley rats with DMH induced CRC	500 mg/kg p.o secoisolariciresinol rich extract of *L. ussitatissimum*—18 weeks	↓ pro-inflammatory markers, ↓ PCNA, ↓ CEA, ↓ mRNA level of CDK4, reduction in hyperplastic cells
Gomides A.F. et al., 2016 [170]	C57 BL6 mice with DMH induced CRC	10% defatted flaxseed meal—15 weeks	Reduction of precancerous lesions in the distal colon
**Sesamol**			
Shimizu S. et al., 2014 [171]	C57/BL6-Apc ^Min/+^ mice	500 pp/8 weeks	↓ pro-inflammatory factors, suppression of intestinal polyps formation

Legend: AOM—azoxymethane, DSS—dextran sulfate sodium; COX2—cycloygenase 2, TNF-α—tumor necrosis factor, PI3K/AKT, Ki-67—proliferative marker, iNOS—nitric oxide synthases, Nf-kB—nuclear factor kappa-light-chain-enhancer of activated B cells, DMH—1,2 dimethylhydrazine, Bcl-2—B-cell lymphoma 2, Bax—Bcl-2associated X protein, PCNA—proliferating cell nuclear antigen, CDK4—cyclin dependent kinase, CEA—carcinogenic embryonic antigen, mir-143—microRNA.

**Table 4:** Summary of *in vivo* experiments regarding CRC and non-phenolic compounds.

Author, Year	Animal Models	Doses and Duration of Administration	Results
**Lycopene**			
Tang F.Y. et al., 2011 [173]	BALB/cAnN-Foxn1 nude mice with CRC induced by inoculation of HT-29 cells	3/6 mg/kg—5 weeks	Significant inhibition of tumor growth
Dias M.C. et al., 2010 [174]	Male Wistar rats with DMH induced CRC	300 mg/kg lycopen + symbiotic (60 mg oligofructose + 50 mg inulin + 10^9^ CFU *Bifidobacteria lactis*)—eight weeks (before/during or after initiation with DMH)	↓ PCNA, ↓ p-53 colonic cells, ↓ AFC, ↓colonic Paneth cells
**Crocin**			
Kawabata K. et al., 2012 [175]	CD1 (ICR) mice with AOM/DSS induced CRC	50, 100, 200 ppm for 15 weeks after initiation of colon cancer	Significant reduction of inflammation and mucosal ulcers, multiplicity of adenocarcinoma
**Oleuropein**			
Giner E. et al., 2016 [176]	C57BL6 mice with AOM/DSS colorectal induced cancer	50 mg/kg or 100 mg/kg—63 weeks	Inhibition of tumor formation, decreased cell proliferation, anti-inflammatory activity
**Capsaicin**			
Caetano B.F.R. et al., 2018 [177]	Male WISTAR rats with DMH induced colorectal cancer	5 mg/kg or 50 mg/kg—four weeks	↓Ki-67, significant ↓ of tumor volume and number of AFC
**Sulforaphane/Organosulfur Compounds**			
Rajendran P. et al., 2015 [178]	Male WT or Nrf2^−/+^ mice with DMH induced CRC	Mice received alternating or daily 400 ppm sulforaphane included in the diet for 25 weeks	Significant reduction in tumor multiplicity only after continuous treatment
**β-Sitosterol**			
Baskar A.A. et al., 2012 [179]	Male albino Wistar rats with DMH induced CRC	5 mg/kg; 10 mg/kg; 20 mg/kg —16 weeks	Significant increase of antioxidant defense system, ↑ GSH, ↓ hyperplasic lesions
**Thymoquinone (TQ)**			
Asfour W. et al., 2013 [180]	Male albino rats with DMH induced CRC	Administration of 10 mg/kg for 10 weeks (in the initiation phase + DMH) and 11 weeks (in the post initiation phase, after induction of cancer)	Chemopreventive effect, significant inhibition of tumor growth (for simultaneously administration in the initiation phase), ↓ PCNA, inhibition of VEGF production
Kortum B. et al., 2015 [181]	Male and female Msh2 ^loxP/loxP^ Villin-Cre mice—transgenic mice that simulate intestinal carcinogenesis	Mice were divided in 5 groups: Group 1—regular chow; group 2—500 mg mesalazine/kg chow, group 3—2500 mg mesalazine/kg chow; group 4—37.5 mg TQ /kg chow; group 5—375 mg TQ/kg chow; treatment for 43 weeks	↓ incidence of tumors dose dependent for TQ; no significant differences between TQ and mesalazine
**Cinnamaldehyde**			
Long M. et al., 2015 [182]	Experimental Nrf2^+/+^ and Nrf2^−/−^ C57BL/6 mice with AOM/DSS induced CRC	Supplementation of diet with 0.5% cinnamaldehyde—11 weeks	Supplementation significantly attenuated colon carcinogenesis only for Nrf2^+/+^ mice; anti-inflammatory and antioxidant effects
**Carvacrol**			
Sivaranjani A. et al., 2016 [183]	Male Albino WISTAR rats with DMH induced CRC	Administration of 20, 40, 80 mg/kg for 16 weeks	Reduced tumor incidence, inhibition of aberrant crypts formation, ↑ in antioxidant defense system, ↓ activity of colonic bacterial enzymes
**Omega-3/Omega-6 Fatty Acids**			
Wang W. et al., 2017 [184]	C57 BL6 mice with CRC induced by inoculation of MC38 CRC cells	Pre-treatment (3 weeks) with DHA diet (omega-6/omega-3 ratio = 1.26:1) and DHA high diet (omega-6/omega-3 ratio = 0.56:1) before tumor initiation (3 weeks) DHA diet—22 g/kg LA, 0.31 g/kg ALA, 17.2 g/kg DHA; DHA high diet—12.5 g/kg—LA, 0.17 g/kg—ALA, 21.9 g/kg DHA	Inhibition of colon growth, modulation of fatty acids profile in colon tumors (↓ARA, ↑EPA, DHA), ↓ EETS
Hao G.W. et al., 2015 [185]	BALB/c nude mice with CRC induced by inoculation of HCT116 colon cancer cells	Ketogenic diet with or without omega-3 fatty acids; supplementation received until tumor volume was 600–700 mm^3^ (45 days)	Delayed tumor growth ↓ tumor vascularity for ketogenic diet supplemented with omega-3 fatty acids
Piazzi G. et al., 2014 [186]	C57BLJ/6J mice with AOM/DSS induced CRC	Effect of 1% eicosapentaenoic free fatty acid on both initiation and progression of carcinogenesis, 105 days	Suppression of tumor development, increase of apoptosis, anti-inflammatory effects, modulation of gut microbiota
Barone M. et al., 2014 [187]	C57BLJ/6J mice with mutation for the *Apc* gene (Apc ^Min/+^)	Supplementation of diet with olive oil and omega-3 fatty acids, 10 weeks	Decrease in polyps number, pro-apoptotic effects
**Dietary Fibers**			
Wang J. et al., 2017 [188]	Athymic male nude mice BALB/c-nu with CRCobtained by inoculation of HT-29 cancer cells	Administration of a polysaccharide from *Lentinus edodes* (0.2 mg/kg; 1 mg/kg; 5 mg/kg) or 20 mg/kg 5-fluorouracil for 21 days after cancer induction	↓ tumor growth, pro-apoptotic effects
Masuda Y. et al., 2013 [189]	Female BALB/c, BALB/c-nude, C3H/HeJ mice inoculated with colon-26 cancer cells	Administration of D fraction (β-glucan) from the maitake mushroom (*Grifola frondolosa*) for 19 days after cancer induction	Significant decrease of tumor growth through systemic immune responses
Pattananandecha T. et al., 2016 [190]	Male Sprague-Dawley rats with AOM induced CRC	Supplementation of diet with 10% inulin for 17 weeks	Reduction of colonic AFC, reduction in bacterial colon enzymes, increase in *Lactobacillus* sp, *Bifidobacteria*
Qamar T.R. et al., 2016 [191]	Male Wistar rats with DMH induced CRC	Administration of galacto-oligosaccharides (76–151 mg), inulin (114 mg) separately or co-administration for 16 weeks	For co-administration significant ↓ in AFC formation and fecal enzyme activities
Hijova E. et al., 2013 [192]	Male and female Sprague-Dawly rats with DMH induced CRC	Supplementation of diet with 80 g inulin/kg food for 28 weeks	Significant ↓ coliform counts and ↑ lactobacilli counts, ↓ fecal enzyme activities, anti-inflammatory effects
Verma A. and Shukla G., 2013 [193]	Male Sprague Dawly rats with DMH induced colorectal cancer	Administration of inulin 10 mg/0.1 mL for a week before initiation of CRC and 6 weeks after initiation	↓ of AFC and nitroreductase/β-glucosidase activity
Stofilova J. et al., 2015 [194]	Male and female Sprague Dawly rats with DMH induced colorectal cancer	Co-administration of oligofructose-enriched inulin preparation (95% fructan chains and 5% monosaccharide and disaccharide) with 10^9^ CFU/mL for 28 weeks	↓ inflammatory process in the jejuna and colon mucosa
Wu W.T. et al., 2014 [195]	Male C57/BL/6J with AOM induced colorectal cancer	Administration of high-fat low fibre diet (1% cellulose) or high 5% fibre diet with konjac glucomannan, inulin, cellulose for 3 weeks before cancer initiation	Konjac glucomannan and inulin have anti-genotoxic effects, increase cecal short chain fatty-acids, up-regulate antioxidant enzymes genes

Legend: AOM—azoxymethane, DSS—dextran sulfate sodium, DMH—1,2 dimethylhydrazine, PCNA—proliferating cell nuclear antigen, AFC—aberrant crypt foci, Ki-67—proliferative marker in the jejum, GSH—glutathione, VEGF C vascular endothelial growth factor, LA—Linoleic acid, ALA—α-linolenic acid, DHA—docosahexaenoic acid, EPA—eicosapentaenoic acid, ARA—arachidonic acid, EETS—epoxyeicosatrienoic acids, CD1 (ICR)—outbred mouse of Swiss origin.

**Table 5:** Summary of the association between CRC risk and phenolic/non-phenolic compounds.

Author, Year	Date/Type of Study	Cases	Control Cases	Dose	OR/HR/RR/IRR/SRR (95%CI)/Observation	Conclusions
**Isoflavones (IF)**						
Akhter M. et al. 2009 [202]	2004–2005/control-study	721; men and women 40–79 years old	697	24.77–62.41 mg IF/day	OR 0.49 (0.27–0.90) to 0.53 (0.28–0.98) p < 0.05	Significant inverse association between high intake of isoflavone consumption and CRC in women
Shin A. et al. 2015 [210]	Case control study/2010–2013	901 men and women	2669	Daidzein 3.20–9.89 mg/day Genistein 3.3–9.7 mg/day Glycitein 0.85–2.44 mg/day	OR Daidzein 1.25 (0.96–1.61) to 0.71 (0.54–0.95) Genistein 1.18 (0.91–1.53) to 0.75 (0.57–1) Glycitein 1.32 (1.03–1.70) to 0.39 (0.25–0.61)	A high intake of isoflavones is significantly associated with decreased risk of CRC in both men and women
Yang G. et al. 2009 [211]	Prospective cohort study /1996–2005	68,412 women 40–70 years old	NA	12.8–21 g soy food intake/day equivalent to 15.1–48.9 mg IF/day	RR Soy food 0.88 (0.67–1.15) to 0.71 (0.53–0.95) Soy isoflavones 0.91 (0.69–1.19) to 0.80 (0.60–1.07)	High intake of soy food products and isoflavones is correlated with reduced incidence in CRC, especially for menopausal women
Ko K. et al. 2018 [205]	Case control study in Korean (1993–2004) and Vietnamese population (2003–2007)	101 (Korean study) 222 cases (Vietnamese study)	391 (Korean study) 226 (Vietnamese study)	Evaluation of plasma IF levels for patients with CRC	OR for genistein 0.67 (0.34–1.31) to 0.50 (0.25–0.98)—Korean patients OR for genistein 0.97 (0.54–1.74) to 0.43 (0.25–0.73)—Vietnamese patients OR for daidzein (Vietnamese patients) 0.84 (0.47–1.49) to 0.48 (0.28–0.82)	Significant inverse correlation between high isoflavones plasma concentrations and reduced colorectal incidence
**Lignans**						
Principi M, et al. 2013 [208]	Randomized double blind placebo-controlled study	30 patients	30 placebo	Supplementation of diet with 750 mg insoluble oat fiber, 50 mg flaxseed dry extract with 20% secoisolariciresinol diglycoside +175 mg milk thistle extract (70% silymarin and 30% silibinin)—60 days prior to colonoscopy	Significant increase in ERβ/ERα ratio and activation of caspases	Modulation of ERβ receptor is important for a chemo-preventive effect
Calabrese C. et al. 2013 [209]	Open study /2012–2013	11 patients with familial adenomatous polyposis with ileal pouch anal anastomosis	NA	5 mg Eviendep® (30% silibinin + 40% secoisolariciresinol diglucoside + indigestible fibers 5% lignin) × 2/day for 3 month	Significant reduction of number and size of polyps with 32% and 51% respectively	Chemo-preventive effect
Zamora-Ros R. et al. 2013 [204]	Case control study /1996–1998	426	401	0.27–0.50 mg lignans/1000 kcal day	RR for Lignans 0.72 (0.47–1.10) to 0.59 (0.34–0.99)	Significant inverse correlation between high intake of lignans and colorectal incidence
**Anthocyanidins**						
Thomasset S. et al. 2009 [212]	Pilot study 2006–2008	15 patients with histological confirmed; 10 patients with colorectal liver metastasis	NA	1.4/2.8/5.6 g of Mirtocyan (a standardized extract rich in anthocyanidins) for 7 days before surgery	Mild decrease of tumor tissue only for 1.4 g	Possible chemo-preventive effects in humans
Wang L. S. et al. 2014 [213]	Randomized double blind placebo control study	14 patients with familial adenomatous polyposis	NA	Group I—7 patients placebo powder (60 g/day) + 2 rectal suppositories (each 720 mg freeze-dried black raspberry extract) Group 2—60 g/day black raspberry freeze dried extract + 2 rectal suppositories—9 month treatment	Reduction of polyps mainly for suppositories Significantly de-methylated regions in adenomas	Regressing of rectal polyps in patients with familial adenomatous polyposis
**Organosulfur Compounds**						
Tanaka S. et al. 2006 [214]	Preliminary double blind randomized clinical trial	37 patients with colorectal adenomas which are removed if the size was > 5 mm	NA	Group I—6 capsules of aged garlic extract (AGE) equivalent to 2.4 mL AGE/day Group II—control (low dose) 6 capsules of AGE equivalent to 0.16 mL AGE/day. Patients are evaluated after 6, 12 months	AGE suppressed colorectal adenomas after 6, 12 months	Chemo-preventive effect in humans
McCullough M. et al. 2012 [215]	CPSII Nutrition cohort 1999–2007	42,824 men 56,876 women	NA	Supplementation of diet with garlic cloves < 1 clove/month; 1–3 cloves/month; 1 clove/week; 2–4 cloves/week; 5–6 cloves/week, 1 clove daily	Protective effect - women HR for 1–3 cloves/week 1.08 (0.86–1.35); 0.95 (0.72–1.26) for 1 clove/week; 0.77(0.58–1.02) for 2–4 cloves/week; 0.74 (0.48–1.13) to 5–6 cloves/week and 0.87 (0.58–1.32) for 1 clove/day	Weak chemo-preventive effect of garlic consumption for women; but not for men
Meng S. et al. 2013 [216]	Cohort study 1984–2008	76,208 women 45,592 men	NA	Administration of garlic cloves < 1 clove/month; 1–3 cloves/month; 1 clove/week; 2–4 cloves/week; 5–6 cloves/week, 1 clove daily	Women HR 1–3 cloves/month 1.11 (0.94–1.31) compared to HR 1.21 (0.94–1.57) for 1 clove/day (*p* = 0.14) Men HR 1–3 cloves/month 0.99 (0.84–1.16) compared to HR 1.03 (0.73–1.45) for 1 clove/day (*p* = 0.99)	No association was found between garlic intake and CRC risk
Satia J. A. et al. 2009 [217]	Cohort study 2000–2002	428	76,084	Administration of garlic pills at least once a week for > 1 year during previous 10 years	HR 1.35 (0.59–1.17) compared to 1.00 (Reference) *p* = 0.04	Significant increase of CRC incidence with garlic administration
**Carotenoids**						
Lu M. S. et al. 2015 [218]	On-going case control study 2010–2013	845	845	Food frequency questionnaire regarding intake of fruits and vegetables rich in carotenoids	α-carotene OR 0.54 (0.42–0.70) to 0.41 (0.31–0.54) β-carotene OR 0.79 (0.61–1.03) to 0.62 (0.48–0.82) lycopene OR 0.66 (0.51–0.85) to 0.45 (0.35–0.60) *p* < 0.001	Significant inverse correlation between carotenoids intake and CRC incidence
Leenders M. et al. 2014 [219]	Cohort study 1992–2000	Colon cancer 898 Rectum cancer 501	898/501	Food frequency questionnaire regarding intake of fruits and vegetables rich in carotenoids and vitamins	For colon cancer β-carotene OR 0.89 (0.67–1.18) to 0.69 (0.52–0.94) Vitamin C OR 0.98 (0.74–1.29) to 0.76 (0.57–1.01) Vitamin E OR 0.88 (0.67–1.16) to 0.99 (0.74–1.33)	Significant inverse correlation between CRC incidence and mainly dietary β-carotene, vitamin C intake
Kabat G. C. et al. 2012 [220]	Large, prospective, multicenter study	88 CRC in post-menopausal women	5389	Analysis of antioxidants from fasting blood samples at baseline, 1/3/6 years follow-up	For CRC β-carotene HR 0.65 (0.39–1.09) to 0.54 (0.31–0.96) For colon cancer β-carotene HR 0.57 (0.32–1.00) to 0.47 (0.25–0.88)	Significant inverse correlation between β-carotene plasma levels and CRC incidence
**Polyunsaturated Fatty Acids**						
Cockbain A. J. et al., 2014 [221]	Phase II double blind randomized placebo control trial 2010–2011	203 patients with CRC liver metastasis	NA	1.Placebo—43 patients 2. Patients receiving 2 g/day of EPA for 30 days prior to surgery, follow-up 18 months after surgery	Significant higher content of EPA in tumor tissues 1.82% compared to 1.30% (for placebo) *p* = 0.0008, decreased PGE2 in tumor tissues, anti-angiogenic activity (*p* = 0.075)	Pre-operative treatment has shown provide post-operative benefit
Song M. et al. 2014 [222]	Study cohort 1984–2008	76,386 women 47,143 men	NA	Administration of fish 15–40 g/day (women) and 16–46 g/day (men), marine fish (0.15–0.30 g/day)	Significant risk of distal colon cancer for both fish intake HR 1.12 (0.85–1.48) to 1.36 (1–1.85) and marine fish HR 1.19 (0.89–1.58) to 1.36 (1.03–1.80) *p* ≤ 0.05	Associated risk between marine fish intake and CRC risk
Sasazuki S. at al. 2011 [223]	Prospective study 1995–2006	827,833 subjects	NA	Food frequency questioners regarding fish intake; marine fish 0.49–2.18 g/day for men and women	Significant Decrease associated with marine fish intake only for men RR 0.97 (0.51–1.83) to 0.35 (0.14–0.88) *p* = 0.05	Chemo-preventive effect of marine fish rich in omega-3 fatty acids
Mocellin M. C. et al. 2013 [224]	Prospective randomized controlled trial 2011–2012	57 patients with CRC undergoing, chemotherapy, only 11 are randomized	NA	1. Control group (*n* = 5). 2. Supplemented group (*n* = 6) with 2g fish oil/day—9 weeks 2 g fish oil = 360 mg/day EPA, 240 mg/day DHA	Significant decrease of C-reactive protein from 18.14 mg/L to 1.14 mg/L (*p* = 0.04) Significant increase of EPA, DHA compared to control group (*p* = 0.014, *p* = 0.019), significant decrease of AA between baseline and 9 weeks follow-up for the supplemented group (*p* = 0.028)	Significant anti-inflammatory effects for patients undergoing chemotheraphy and increase for plasma fatty acid profile
Mocellin M. C. et al. 2016 [225]	Meta-analysis of Nine trials	475 patients with CRC	NA	Supplementation of diet with omega-3 fatty acids or administration of 0.2 g/kg fish oil parenterally at post-operative period Patients undergoing chemotherapy supplementation with 0.6 g/day EPA+DHA -9 weeks	Significant decrease of IL-6 (*p* = 0.024) and increase of albumin (*p* = 0.014) Supplementation of EPA+ DHA during chemotherapy significantly reduced CRP concentration (*p* = 0.017) and CRP/albumin ratio (*p* = 0.016)	Use of omega-3 fatty acids have benefits, especially for inflammatory markers in CRC patients
Sorensen L. S. et al. 2014 [226]	Randomized double blind placebo controlled trial	148 patients awaiting for CRC surgery	NA	1. Control group 2. Supplemen-tation group with 2 g EPA + 2 g DHA for 7 days before and 7 days after surgery	Pre-operative treatment with omega-3 fatty acids determined a significant increase of EPA, DHA levels in granulocytes and a significant decrease of AA (*p* < 0.001) compared to control group	Potential immune-stimulatory effects and prevention of post-operative infections
Ma C. J. et al. 2015 [227]	Prospective randomized double-blind study 2009–2010	99 patients with gastric and CRC	NA	1. Control group 2. Supplementation group with a lipid emulsion containing soybean oil (80–100 g/L), medium chain triglycerides (100 g/L) and PUFA (linoleic acid—38–58 g/L, α-linolenic acid—4–11 g/L) for 7 days after surgery	There are no significant differences regarding inflammatory markers between the control and the supplementation group. Significant positive effect on lipid markers (*p* < 0.05)	Improvements only in lipid metabolism
**Dietary Fiber**						
Holscher H. D. et al. 2015 [228]	Prospective Randomized double-blind placebo 3-period controlled study	30 heathy patients	NA	1. Placebo group 2. Administration of 5 g or 7.5 g inulin for 21 days with 7 days wash-out between periods	Significant increase of *Bifidobacterium sp.* (*p* < 0.001), significant decrease of *Ruminococcus sp.* and *Desulfovibrio sp.* (*p* < 0.01); dietary intake was positively associated with fecal butyrate (*p* = 0.005)	Beneficial changes in gastro-intestinal microbiota are correlated with decreased CRC incidence
Limburg P et al, 2011 [229]	Randomized phase II clinical trial 2006–2008	85 patients with aberrant crypt foci ≥ 5 at baseline	NA	1. Control group 2. Atorvastatin 20 mg/day 3. Sulindac 150 mg x 2/day 4. Oligo-fructose enriched inulin 6 g powder x 2/day for 6 months	All treatment didn’t provide a significant decrease in AFC number and size	No association was found between inulin intake and CRC
Mehta RS et al, 2018 [230]	Prospective cohort study 1980–2012	121,700 females 51,529 males	NA	Food frequency questionnaires regarding dietary fiber intake	High intake of fiber was associated with a low risk of *Fusobacterium nucleatum* positive CRCs HR 0.54 (0.33–0.89) to 0.40 (0.24–0.67)	Intestinal microbiota plays an important role in mediating the association between consumption of high amount of dietary fiber/whole grains and CRC incidence
Ben Q et al, 2014 [231]	Meta-analysis of 20 studies (case-control, cohort)	10,984 patients with colorectal adenoma	NA	Administration of 10 g/day fibers	SRR for dietary fiber are 0.72 (0.63–0.83) in a high vs low intake, inverse association between total fiber intake and CRC risk SSR 0.66 (0.56–0.77)	Chemopreventive effect of dietary fiber
Hansen L et al, 2012 [232]	Cohort study, 1997–2008	108,081 patients	NA	Administration of fiber 16–28 g/day for men and 15–24 g for women	Significant inverse correlation between CRC incidence and dietary fiber intake for men IRR 0.93 (0.68–1.26) to 0.55 (0.38–0.79)	Chemopreventive effect of dietary fiber
Kunzman A et al, 2015 [233]	Cohort study 1993–2009	2036 patients	15,976	Administration of fiber 9.9–12.8 g/1000 kcal/day from fruits/vegetables	Significant inverse correlation between dietary fiber intake and distal colon or rectal adenoma in men OR 0.88 (0.75–1.04) to 0.76 (0.63–0.91) *p* = 0.003	Chemopreventive effect of dietary fiber against CRC
Murphy N et al, 2012 [234]	On-going multicentre prospective cohort study 1992–2000	477,312 patients	NA	Administration of dietary fiber 16.4–28.5 g/day	Significant inverse correlation between dietary intake and *colon-distal cancer* HR 0.90(0.75–1.07) to 0.70 (0.53–0.92) *p* = 0.021 colon proximal cancer HR 0.93 (0.78–1.1) to 0.86 (0.69–1.07) *p* = 0.16 rectum cancer HR 1 (0.87–1.17) to 0.79 (0.65–0.96) *p* = 0.012	Chemopreventive effect of dietary fiber against CRC
Mathers JC et al, 2012 [235]	Randomized control trial	937 eligible patients with Lynch syndrome	NA	1. 463 patients received 30 g resistant starch/day 2. 455 patients—resistant starch placebo 3. 19 patients 600 mg aspirin/day—29 months	No significant effect of resistant-starch administration on cancer development IRR resistant starch vs resistant starch placebo 1.15 (0.66–2.00) *p* = 0.61	No detectable effect on cancer development

Legend: HR—hazard ratio; RR—relative risk; OR—odds ratio; IRR—incidence rate ratio; SRR—summary relative risk; NA, not applicable.

**Table 6:** Summary of the bioavailability of the dietary compounds.

Compound of Interest	Source	Bioavailability	In Vivo Studies	Ref.
Curcumin	Turmeric	- poor absorption, rapid metabolism, rapid elimination - enhanced by piperine with 2000% - better results on animal experiments	- 2g/kg of curcumin in rats → *C_max_* = 1.35 ± 0.23 μg/mL in 0.83 h - 2 g/kg of curcumin in human subjects → *C_max_* = 0.006 ± 0.005 μg/mL	[120,248]
EGCG	Green tea	- poor absorption - alkaloids, vitamins, proteins and fish oil improve absorption - air contact oxidation, metal ions like Ca^2+^ and Mg^2+^ and milk reduce absorption	- one oral dose of EGCG half-time = 3.4 ± 0.3 h	[249]
Resveratrol	Grapes	- poor absorption - bioavailability increased by a liquid micellar formulation	- 500 mg of Vineatrol → *C_max_* of trans-resveratrol = 10.6 fold higher - no detection of trans-ε-viniferin in plasma or urine	[250]
Quercetin	Onion Apples Beans Broccoli	- better bioavailability of quercetin glucoside	- 100 mg quercetin → absorption of quercetin glucoside = 3–17%	[251]
Genistein	Soy	- lower bioavailability in vivo - total genistein—a better bioavailability than genistein aglycone	- 20 mg/kg of genistein in FVB mice → genistein aglycone bioavailability = 23.4% - soy protein feeding Balb/c mice females → bioavailability of total genistein = 90% → bioavailability of genistein aglycone = < 15%	[252]
Anthocyanins	Bilberries	- poor bioavailability - 30% of amthocyanins are stable in the upper intestine for 8 h.	- bioavailability of anthocyanins from bilberries → ↑ amount of anthocyanins and degradants in the heathy compared to ileostomists group	[253]
Proantho-cyanidin	Apples Grapes Green tea	- oligomeric flavonoids with limited bioavailability	- ad libitum diet of grape seed extract in lab rats → the presence of PAC in the colonic contents - 11% of PAC—present in the feces	[254]
Capsaicin	Chilli	- low bioavailability - capsaicin was absorbed into intestinal tissues, jejunum and serosal fluid	- 1 mM of capsaicin in rats → absorption = 50% in the stomach, 80% in the jejunum and 70% in the ileum.	[255]
Piperine	Black pepper	- insoluble in water with a low bioavailability - used in clinical assays single or as an enhancer for other dietary agents - improved the bioavailability of resveratrol, curcumin and lycopene	- resveratrol + piperine in mice → piperine enhanced the bioavailability of resveratrol with 229% - curcumin + piperine in rats and human subjects → piperine enhanced bioavailability of curcumin with 2000%	[256,257]
Aliicin	Garlic	- poor bioavailability	- administration of garlic/ pure allicin → no detection in urine or blood	[258]
